# CD4^+^ anti-TGF-β CAR T cells and CD8^+^ conventional CAR T cells exhibit synergistic antitumor effects

**DOI:** 10.1016/j.xcrm.2025.102020

**Published:** 2025-03-18

**Authors:** Diwei Zheng, Le Qin, Jiang Lv, Meihui Che, Bingjia He, Yongfang Zheng, Shouheng Lin, Yuekun Qi, Ming Li, Zhaoyang Tang, Bin-Chao Wang, Yi-Long Wu, Robert Weinkove, Georgia Carson, Yao Yao, Nathalie Wong, James Lau, Jean Paul Thiery, Dajiang Qin, Bin Pan, Kailin Xu, Zhenfeng Zhang, Peng Li

**Affiliations:** 1 China-New Zealand Joint Laboratory on Biomedicine and Health, National Key Laboratory of Immune Response and Immunotherapy, Guangdong Provincial Key Laboratory of Stem Cell and Regenerative Medicine, GIBH-HKU Guangdong-Hong Kong Stem Cell and Regenerative Medicine Research Centre, GIBH-CUHK Joint Research Laboratory on Stem Cell and Regenerative Medicine, Institute of Drug Discovery, Guangzhou Institutes of Biomedicine and Health, Chinese Academy of Sciences, Guangzhou, China; 2 Centre for Regenerative Medicine and Health, Hong Kong Institute of Science & Innovation, Chinese Academy of Sciences, Hong Kong SAR, China; 3 Department of Radiology, Translational Provincial Education Department Key Laboratory of Nano-Immunoregulation Tumor Microenvironment, the Second Affiliated Hospital of Guangzhou Medical University, Guangzhou, China; 4 Blood Disease Institution, Department of Hematology, the Affiliated Hospital of Xuzhou Medical University, Xuzhou Medical University, Xuzhou, Jiangsu, China; 5 Department of Surgery of the Faculty of Medicine, the Chinese University of Hong Kong, Hong Kong SAR, China; 6 Guangdong Zhaotai Cell Biology Technology Ltd., Foshan, China; 7 Guangdong Lung Cancer Institute, Guangdong General Hospital (GGH) & Guangdong Academy of Medical Sciences, Guangzhou, China; 8 Cancer Immunotherapy Programme, Malaghan Institute of Medical Research, Wellington, New Zealand; 9 Guangzhou Laboratory, Guangzhou, China; 10 The Fifth Affiliated Hospital of Guangzhou Medical University, Guangzhou, China

**Keywords:** TGFβ1, CAR T, T cell exhaustion, glypican-3, mesothelin, mitochondrial fission, SMAD4

## Abstract

Transforming growth factor (TGF)-β1 restricts the expansion, survival, and function of CD4^+^ T cells. Here, we demonstrate that CD4^+^ but not CD8^+^ anti-TGF-β CAR T cells (T28zT2 T cells) can suppress tumor growth partly through secreting Granzyme B and interferon (IFN)-γ. TGF-β1-treated CD4^+^ T28zT2 T cells persist well in peripheral blood and tumors, maintain their mitochondrial form and function, and do not cause *in vivo* toxicity. They also improve the expansion and persistence of untransduced CD8^+^ T cells *in vivo*. Tumor-infiltrating CD4^+^ T28zT2 T cells are enriched with TCF-1^+^IL7R^+^ memory-like T cells, express NKG2D, and downregulate T cell exhaustion markers, including PD-1 and LAG3. Importantly, a combination of CD4^+^ T28zT2 T cells and CD8^+^ anti-glypican-3 (GPC3) or anti-mesothelin (MSLN) CAR T cells exhibits augmented antitumor effects in xenografts. These findings suggest that rewiring TGF-β signaling with T28zT2 in CD4^+^ T cells is a promising strategy for eradicating solid tumors.

## Highlights

• Anti-TGF-β CAR prevents mitochondrial fission in CD4 T cells upon TGF-β treatment
• CD4 but not CD8 anti-TGF-β CAR T cells are effective for tumor growth inhibition
• CD4 anti-TGF-β CAR T cells exhibit memory T cell features and prevent T cell exhaustion
• CD4 anti-TGF-β CAR T cells and CD8 CAR T cells synergistically inhibit tumor growth

## Graphical abstract

**Figure:**
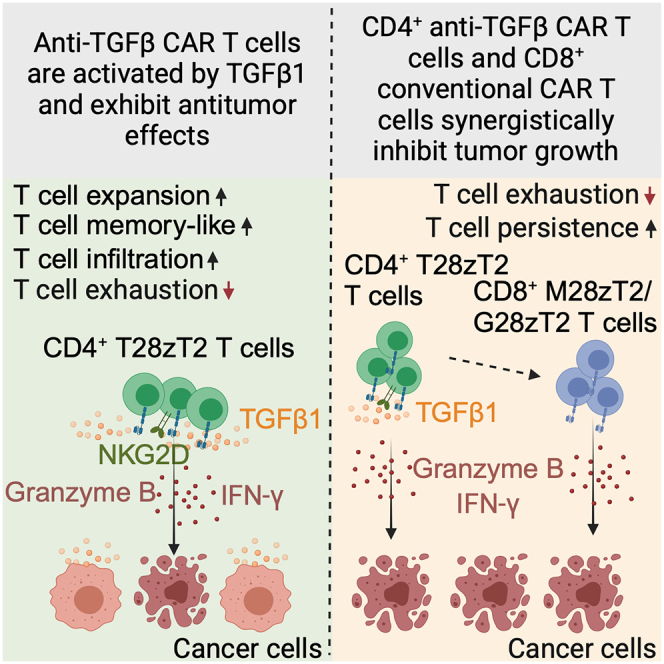

## Teaser

In this study, Zheng et al. report that rewiring TGF-β signaling with anti-TGF-β CAR can promote T cell expansion, augment T cell infiltration, and prevent T cell exhaustion and thus is a promising strategy for an improved efficacy of CD4^+^ T cells in eradicating solid tumors.

## Introduction

CD4^+^ T cells are antitumor effectors via coordinating innate and antigen-specific immune responses and killing cancer cells.^1^^,^^2^ CD4^+^ T cells cooperate with macrophages and monocytes to induce inflammatory cell death of tumors in the absence of CD8^+^ T cells.^3^ Cytotoxic CD4^+^ T cells, a subtype of CD4^+^ T cells expressing granzymes, granulysin (GNLY) and perforin, are detected in tumors.^4^^,^^5^ Recent studies report that CD4^+^ chimeric antigen receptor (CAR) T cells directly kill cancer cells by producing interferon (IFN)-γ^6^ and enhance the persistence and efficacy of CD8^+^ CAR T cells.^7^ However, the antitumor effects and helper functions of CD4^+^ T cells can be suppressed by external factors such as PD-L1^8^ and transforming growth factor (TGF)-β1^9^ and are regulated by mitochondrial dynamics.^10^

TGF-β1 is important for development and homeostasis.^11^ TGF-β1 activation results in phosphorylation of TGF-βR1.^12^ Pathological TGF-β signaling promotes metastasis and evasion of immune surveillance.^13^ TGF-β1 represses the antitumor effects of T cells by inducing PD-1 expression.^14^ It also promotes the differentiation and proliferation of T regulatory cells (Tregs)^15^ and cancer-associated fibroblasts (CAFs).^11^ Thus, TGF-β signaling is an attractive target for cancer treatment.

There are multiple strategies to inhibit TGF-β signaling in immunotherapies, including anti-TGF-β1 antibody treatment,^16^ overexpression of the dominant-negative TGF-β type II receptor (DNTR),^17^ and ablation of TGF-βRII.^18^^,^^19^^,^^20^ Another strategy is rewiring of immune-suppressive TGF-β-SMAD signaling into T cell activation signaling via an anti-TGF-β CAR, which promotes T cell expansion and cytokine production.^21^ Nevertheless, it remains unclear which compartment of anti-TGF-β CAR T cells mainly contributes to antitumor effects and how this compartment inhibits tumor growth.

Here, we characterized the antitumor activity of anti-TGF-β CAR T cells and found that their CD4^+^ but not CD8^+^ compartment induced the death of cancer cells *in vitro* and robustly suppressed the growth of various tumor models. These CD4^+^ cells were protected from the mitochondrial fission of TGF-β1 treatment and showed no *in vivo* toxicity. We then combined CD4^+^ anti-TGF-β CAR T cells with CD8^+^ anti-mesothelin (MSLN) or anti-GPC3 CAR T cells to treat solid tumors and finally investigated the effects of CD4^+^ anti-TGF-β CAR T cells on conventional CD8^+^ CAR T cells in xenografts.

## Results

### Anti-TGF-β CAR T cells suppress tumor growth and promote T cell expansion *in vivo*

**Figure 1:**
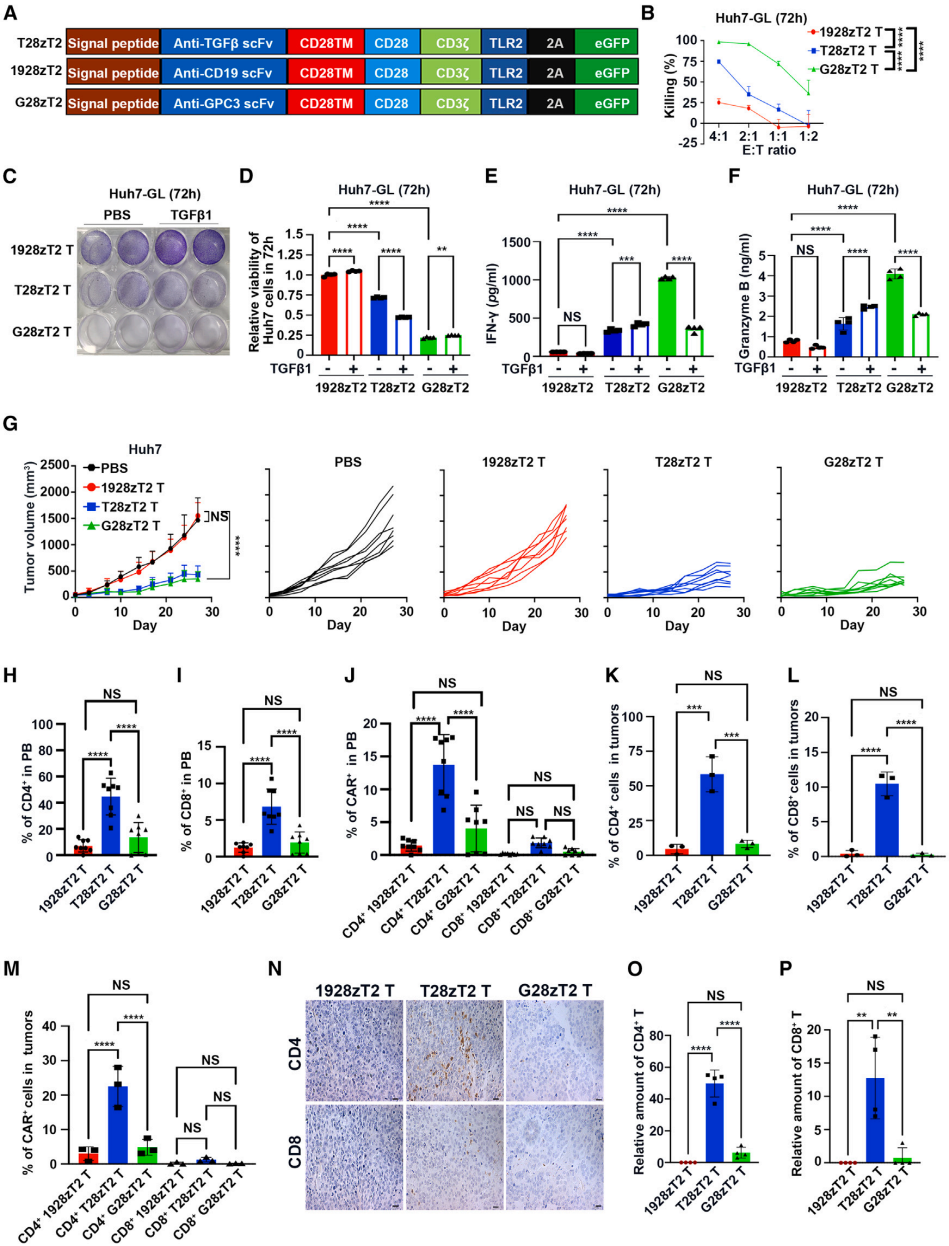
Anti-TGF-β CAR T cells reduced tumor growth and promoted T cell expansion *in vivo* (A) Anti-TGF-β CAR vector (T28zT2), anti-GPC3 CAR vector (G28zT2), and anti-CD19 CAR vector (1928zT2) based on an anti-TGF-β scFv (US20140127230A1), anti-GPC3 scFv (GC33), or anti-CD19 scFv (FMC63), respectively. All contained expression cassettes encoding a human CD8 leader signal peptide, CD28, CD3ζ, and TLR2 signaling domain along with eGFP using 2A self-cleaving peptide (2A). The eGFP expression was used to monitor CAR-transduced cells. (B) The percentage of Huh7 cells with 1928zT2, G28zT2, or T28zT2 T cell-induced lysis after 72 h; data are the mean percentage of tumor cell-specific lysis ± SEM values; *n* = 3 independent experiments; two-way ANOVA with Tukey’s multiple comparisons test; ∗∗∗∗*p* ≤ 0.0001. (C–F) A total of 4 × 10^5^ 1928zT2, T28zT2, or G28zT2 T cells were treated with PBS or TGF-β1 (10 ng/mL) for 15 min. These T cells were then cocultured with 1 × 10^5^ Huh7 cells for 72 h at a 4:1 effector (E):target (T) ratio in 12-well round bottom plates for 72 h. Supernatants were harvested and analyzed with a multiplex immunoassay to determine the concentrations of the indicated cytokines. (C) Representative crystal violet images of Huh7 cells with 1928zT2, T28zT2, or G28zT2 T cells-induced lysis after 72 h. Quantification of residual tumor cells (D) and summary of IFN-γ (E) and Granzyme B (F) released by CAR T cells (from 4 independent experiments); data are the mean ± SEM values; one-way ANOVA with Tukey’s multiple comparisons test; ∗∗*p*≤ 0.01, ∗∗∗*p*≤ 0.001, ∗∗∗∗*p*≤ 0.0001. (G) Eight-week-old male NSI mice were inoculated subcutaneously with 2 × 10^6^ Huh7 cells into the right flanks. A total of 5 × 10^6^ CAR T cells or PBS was injected peritumorally when the xenograft volume was ∼50 mm^3^ (day 0). The majority of mice exhibited severe graft-versus-host disease (GVHD) symptoms, halting animal experiments on day 27. Growth curves of Huh7 tumors in NSI mice post-infusion of T28zT2, G28zT2, and 1928zT2 T cells or PBS treatment (*n* = 8 mice/group); data are the mean ± SD values; two-way ANOVA with Tukey’s multiple comparisons test; ∗∗∗∗*p*≤ 0.0001. (H–J) The percentages of CD4^+^ (H), CD8^+^ (I), CAR^+^(GFP^+^) CD4^+^ (square, J), and CAR^+^CD8^+^ (triangle, J) T cells (*n* = 8 mice/group) of all nucleated cells in murine peripheral blood (PB) were determined by flow cytometry for the T28zT2, G28zT2, and 1928zT2 groups on day 27; data are the mean ± SD values; one-way ANOVA with Tukey’s multiple comparisons test; ∗∗∗∗*p*≤ 0.0001. (K–M) The percentages of CD4^+^ (K), CD8^+^ (L), and CAR^+^ (M) T cells in all nucleated cells from Huh7 tumors in the 1928zT2, T28zT2, and G28zT2 groups on day 27 determined by flow cytometry (*n* = 3 mice per group). Data are shown as the mean ± SD values; one-way ANOVA with Tukey’s multiple comparisons test; ∗∗∗*p*≤ 0.001, ∗∗∗∗*p*≤ 0.0001. (N–P) Representative images of CD4^+^ (N, top) and CD8^+^ (N, bottom) T cells (brown) in Huh7 tumors from the T28zT2, G28zT2, and 1928zT2 groups on day 27. The frequencies of CD4^+^ (O) and CD8^+^ T cells (P) were calculated by ImageJ software (*n* = 4 mice per group). Scale bar, 20 μm. Data are shown as the mean ± SD values; one-way ANOVA with Tukey’s multiple comparisons test; ∗∗*p*≤ 0.01, ∗∗∗∗*p*≤ 0.0001. See also in Figures S1 and S2.

We established third-generation CAR vectors containing an anti-TGF-β single-chain variable fragment (scFv), a human CD28 transmembrane domain (CD28TM) and an endodomain, a human CD3ζ signaling domain, and an enhanced GFP (eGFP) used as a CAR^+^ cell tag with (T28zT2) or without (T28z) an intracellular domain of human Toll-like receptor (TLR)2 that improve antitumor activity of CAR T cells^22^^,^^23^^,^^24^^,^^25^ (Figures 1A and S1A). Compared with TLR2-lacking T28z T cells, these T28zT2 T cells produced higher amounts of interleukin (IL)-2 and IFN-γ upon TGF-β1 treatment (Figures S1B and S1C), suggesting that incorporation of the TLR2 domain improved cytolytic cytokine production of anti-TGF-β CAR T cells. We then generated an anti-TGF-β CAR vector without a human CD3ζ signaling domain (T28T2). Unlike these T28T2 Jurkat cells, T28zT2 Jurkat cells increased CD69 expression upon TGF-β1 treatment (Figure S1D), suggesting that a CD3ζ signaling domain is required for the responsiveness to TGF-β1 treatment. Therefore, we used the T28zT2 vector for further investigation.

Upon TGF-β1 treatment, the phosphorylation of SMAD2/3, which are induced when TGF-β1 binds to TGF-βR1,^12^ was inhibited in T28zT2 T cells compared to 1928zT2 T cells (Figure S1E). T28zT2 T cells also upregulated CD25 and CD69 expression upon TGF-β1 treatment, compared to that of 1928zT2 T cells (Figure S1F). TGF-β1 is highly expressed in Huh7, a hepatocellular carcinoma (HCC) cell line (Figure S1G). We generated T28zT2 T cells, 1928zT2 T cells as a negative control, and anti-GPC3 (G28zT2) T cells targeting GPC3, which is highly expressed in HCC,^26^ as a positive control (Figure 1A). There were no significant differences on the lysing capacity of T28zT2 T cells and 1928zT2 T cells against Huh7-GL cells that expressed GPC3, eGFP, and luciferase, with or without TGF-β1 treatment after 24 h, though T28zT2 T cells increased IFN-γ and Granzyme B secretion upon TGF-β1 treatment (Figures S1H–S1J). In contrast, G28zT2 T cells efficiently lysed Huh7-GL cells, but their lysing capacity was reduced upon TGF-β1 treatment (Figure S1H). IFN-γ and Granzyme B production from G28zT2 T cells also decreased sharply upon TGF-β1 treatment (Figures S1I and S1J). Notably, T28zT2 T cells could lyse Huh7-GL cells after coculture for 72 h (Figure 1B). Furthermore, at this time point, TGF-β1 treatment increased lysing (Figures 1C and 1D) and IFN-γ and Granzyme B production (Figures 1E and 1F) capacities of T28zT2 T cells but decreased those of G28zT2 T cells, suggesting that T28zT2 T cells require a longer duration to effectively suppress Huh7 expansion.

We next compared the antitumor effects of T28zT2 T cells in Huh7 xenografts, which were established in immunodeficient non-obese diabetic (NOD)-severe combined immunodeficiency (SCID)-*IL-2Rg*^−/−^ (NSI) mice that lack T, B, and natural killer (NK) cells.^27^ T28zT2 and G28zT2 T cells, but not 1928zT2 T cells, suppressed the growth of Huh7 cells (Figure 1G). The CD4/CD8 human T cell ratios collected from peripheral blood samples of xenograft-bearing mice in the T28zT2 group were increased compared to those in the 1928zT2 and G28zT2 groups (Figures S1K–S1N). Remarkably, the percentages of CD4^+^, CD8^+^, and CAR^+^CD4^+^ T cells from murine peripheral blood (Figures 1H–1J), spleen (Figures S1O and S1P), and tumor (Figures 1K–1P) samples of the T28zT2 group were significantly higher than those of the G28zT2 and 1928zT2 groups. Of interest, there were also more CAR^+^ tumor-infiltrating T cells that expressed GFP and more apoptotic cells that were cleaved-caspase-3 (CC3)-positive in tumors from the T28zT2 group than in tumors from the G28zT2 and 1928zT2 groups (Figures S1Q–S1S). A previous study suggested that TGF-β1 induces the differentiation of alpha-smooth muscle actin (αSMA)-positive CAFs,^28^ which promote tumor metastasis^29^ and immune evasion.^30^ Indeed, the percentages of αSMA^+^ CAFs in tumors from the T28zT2 group were significantly lower than those from the 1928zT2 and G28zT2 groups (Figures S1T and S1U). Possibly, T28zT2 T cells blocked CAF differentiation indirectly by depriving them of TGF-β1 in tumors. T28zT2 T cells also demonstrated effective suppression of tumor growth in two HCC patient-derived xenograft (PDX) models (Figures S2A and S2B), and both T28zT2 T cells and anti-MSLN CAR T cells (M28zT2 T cells) effectively inhibited tumor progression in HeLa xenografts (Figures S2C and S2D). These results collectively demonstrate that anti-TGF-β CAR T cells efficiently suppressed the growth of multiple types of tumors and promote T cell expansion *in vivo.*

T cells expressing the DNTR exhibit antitumor effects.^19^^,^^20^ We thus compared their efficacies *in vivo*. T cells expressing T28zT2, DNTR, or 1928zT2 were infused into immunodeficient NSI mice bearing Huh7 cells (Figures S2E and S2F). Of note, T28zT2 T cells displayed a better antitumor effect in Huh7 models (Figure S2G) than DNTR T cells that did not express the specific CAR. In addition, the percentages of peripheral blood-derived T cells in the T28zT2 and DNTR groups were significantly higher than those of the 1928zT2 group (Figure S2H). These results suggest that T28zT2 T cells were more effective than DNTR T cells at *in vivo* tumor killing, possibly as T28zT2 T cells rewired TGF-β signaling further into CAR signaling, promoting their effector function, compared with DNTR T cells.

### CD4^+^ anti-TGF-β CAR T cells exhibit memory T cell phenotypes and prevent T cell exhaustion *in vivo*

**Figure 2:**
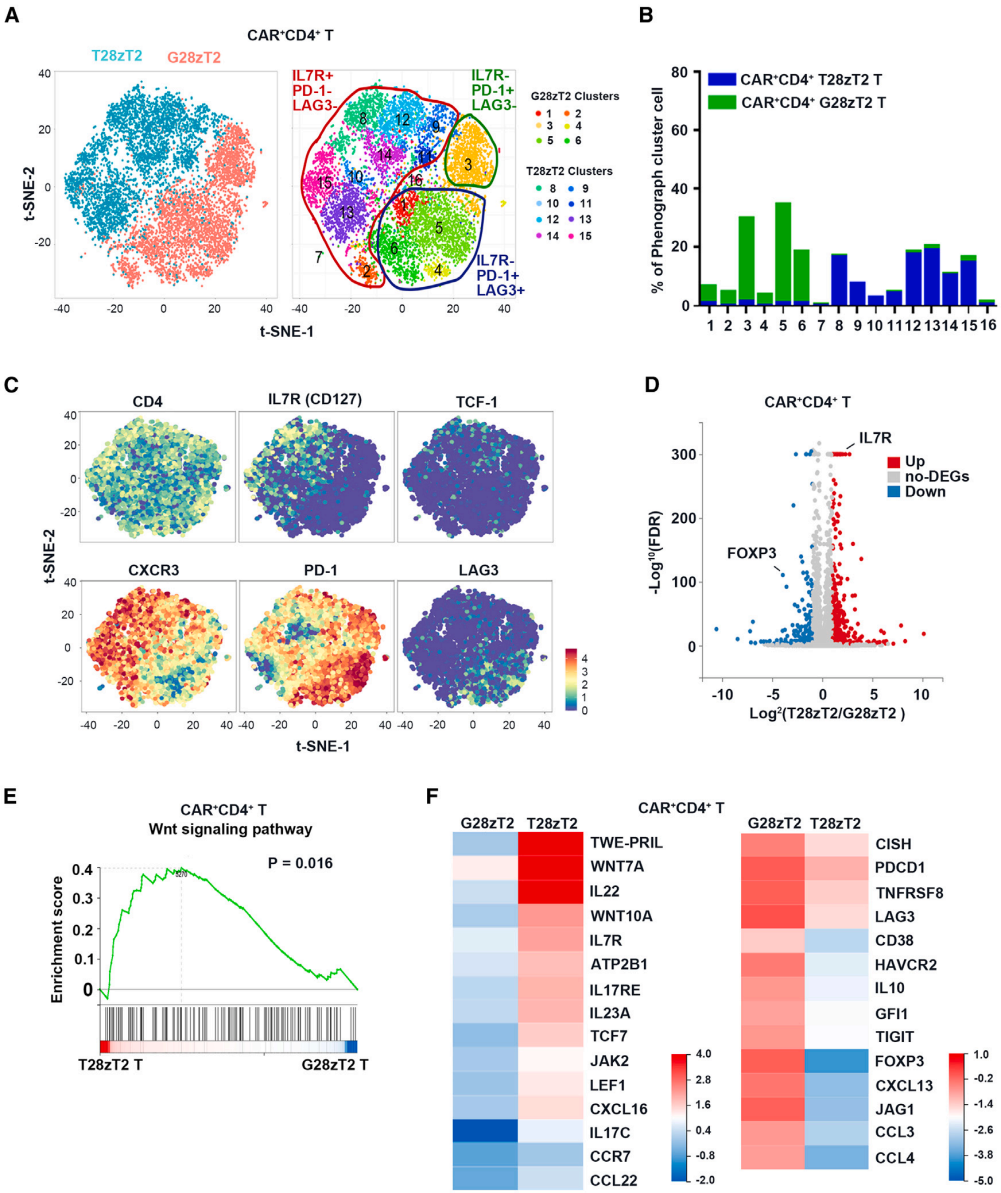
CD4^+^ anti-TGF-β CAR T cells exhibit memory-like T cell phenotypes in xenografts (A) 2D projection of the sample distribution (left) and subclusters (right) of purified splenic CAR^+^(GFP^+^) CD4^+^ T cells from the T28zT2 group (blue) and G28zT2 (red) group using t-SNE. T28zT2 clusters contain clusters 8–15, defined as IL7R^+^PD-1^−^LAG3^−^ T cell subsets; G28zT2 clusters contain clusters 1–6, defined as IL7R^−^PD-1^+^LAG3^+^ T cell subsets or IL7R^−^PD-1^+^LAG3^−^ T cell subsets. (B) PhenoGraph cluster distribution comparing CAR^+^CD4^+^ T cells between the T28zT2 group (blue) and G28zT2 (green) group. (C) Differences in gene expression between the T28zT2 and G28zT2 groups of individual purified splenic CAR^+^(GFP^+^) CD4^+^ T cells in the t-SNE projection, including CD4, IL7R (CD127), TCF-1, CXCR3, PD-1, and LAG3. (D) Volcano plot of DEGs showing upregulated (red) and downregulated (blue) DEGs and non-DEGs (gray) identified by RNA-seq in T28zT2 CAR^+^CD4^+^ T cells compared to G28zT2 cells. Adjustment for the false discovery rate (FDR) results in an adjusted *p* value called the *q* value. The *y* axis shows the significance value after −log_10_ transformation of the FDR (−Log_10_(FDR)). The x axis shows the fold difference threshold between the T28zT2 and G28zT2 groups (Log_2_(T28zT2/G28zT2)). (E) GSEA of the Wnt signaling pathway (*p* = 0.016) in CAR^+^CD4^+^ T cells. From left to right, the genes in the rank-ordered list are enriched in the T28zT2 and G28zT2 groups. (F) Heatmap of DEGs involved in T cell differentiation, exhaustion, and activation in CAR^+^CD4^+^ cells identified in comparisons between the T28zT2 and G28zT2 groups. Cutoff: absolute log2 (fold change) ≥ 1; adjusted *p* value ≤ 0.05. See also in Figure S3.

To characterize the effects of T28zT2 on CD4^+^ T cells, CAR^+^CD4^+^ T cells from the spleen of Huh7-xenografted mice in the T28zT2 and G28zT2 groups were purified and subjected to cytometry by time-of-flight (CyTOF) analysis. Based on t-distributed stochastic neighbor embedding (t-SNE), CAR^+^CD4^+^ T28zT2 T cells were classified into 8 clusters (clusters 8–15) and separated from CAR^+^CD4^+^ G28zT2 T cells, which localized in clusters 1–6 (Figures 2A and 2B). Some T cells in clusters 8 and 12 expressed TCF-1 and IL7R (Figures 2A and 2C), markers of memory T cells.^31^^,^^32^^,^^33^^,^^34^^,^^35^ In contrast, the T cells in clusters 1 and 4–6 exhibited very low expression of these markers but high levels of exhausted T cell markers such as PD-1 and LAG3 (Figure 2C).

We then investigated the transcriptomes of CAR^+^CD4^+^ T28zT2 T cells in the spleen of Huh7 xenografts using bulk RNA sequencing (RNA-seq). Compared to CAR^+^CD4^+^ G28zT2 T cells, CAR^+^CD4^+^ T28zT2 T cells exhibited 717 differentially expressed genes (DEGs) (Figure 2D; Tables S1 and S2). Gene set enrichment analysis (GSEA) further showed the enrichment of Wnt signaling-related genes, which promote memory T cell formation,^32^ in CAR^+^CD4^+^ T28zT2 T cells (Figure 2E). Furthermore, heatmap analysis revealed that, compared to CAR^+^CD4^+^ G28zT2 T cells, Wnt signaling-related genes, such as *WNT10A*, *WNT7A*, *TCF7*, and *LEF1*, were upregulated (Figure 2F), whereas *LAG3*, *TIGIT*, and *PDCD1* were downregulated (Figure 2F) in CAR^+^CD4^+^ T28zT2 T cells. Interestingly, the expression of Th17- and Th22-related cytokines, including *IL17C*, *IL17RE*,^36^ *IL22*,^37^ and *IL23A*,^38^ was increased in CAR^+^CD4^+^ T28zT2 T cells (Figure 2F), while the expression of Treg-associated genes, including *FOXP3*,^39^^,^^40^ *IL10*, and *JAG1*,^41^ and the expression of CD38, a nicotinamide adenine dinucleotidehydrolase (NADase) that inhibits oxidative phosphorylation (OXPHOS),^42^ were decreased compared to CAR^+^CD4^+^ G28zT2 T cells (Figure 2F). Therefore, CAR^+^CD4^+^ T28zT2 T cells exhibit memory-like T cell phenotypes *in vivo*.

To further dissect the subsets of tumor-infiltrating lymphocytes (TILs) in T28zT2 and G28zT2 groups, we purified CD3^+^ cells from tumor single-cell suspensions in the T28zT2 and G28zT2 groups and performed CyTOF analysis. Based on t-SNE, these CD3^+^ cells from the two groups were classified into 8 clusters (Figure S3A). Clusters 1, 2, and 4 were mainly composed of CD3^+^ cells from the T28zT2 group, while most cells in clusters 3, 6, 7, and 8 were from the G28zT2 group (Figure S3B). Similar to spleen-derived T28zT2 T cells (Figure 2C), CD3^+^ cells from the T28zT2 group expressed a high level of CXCR3 (Figure S3C), which is suppressed by TGF-β1^43^ and positively correlated with elevated intratumoral T cell infiltration^44^ and the efficacy of anti-PD-1 therapies.^45^ G28zT2 CD3^+^ cells lacked this high CXCR3 expression but expressed PD-1, T-cell immunoreceptor with Ig and ITIM domains (TIGIT), and TIM-3 at higher levels than those from the T28zT2 group (Figure S3C). However, T28zT2 CD3^+^ cells did not increase IL7R expression (Figure S3C), compared with those from the G28zT2 group. The IL7R expression differences in spleen-derived T cells (Figure 2C) and tumor-infiltrating CD3^+^ cells (Figure S3C) were probably due to different original microenvironments. We also performed RNA-seq analysis on these purified tumor-infiltrating CD3^+^ cells and found that those from the T28zT2 group exhibited upregulation of genes related to pathways involved in T cell receptor (TCR) signaling, NK cell-mediated cytotoxicity, IFN-γ-mediated signaling, and the regulation of T cell proliferation (Figures S3D–S3G; Tables S3 and S4). In addition, cytotoxic genes (*ICOS*, *IFNG*, *LCK*, *NKG2D*, *TRAIL*, *FASLG*, *TNFSF8*, *NCR3*, etc.) and memory T cell-related genes, such as *TCF7* and *IL7R*, were upregulated in CD3^+^ cells from the T28zT2 group compared to CD3^+^ cells from the G28zT2 group (Figure S3H).

We then purified CAR^+^CD4^+^ T28zT2 and CAR^+^CD4^+^ G28zT2 T cells from Huh7 xenograft tumors and evaluated their cytokine production, cytotoxicity, and proliferation upon TCR activation. CAR^+^CD4^+^ T28zT2 T cells lysed Huh7 cells more efficiently (Figure S3I), secreted higher levels of IFN-γ (Figure S3J) but lower levels of Granzyme B (Figure S3K), and exhibited more robust expansion (Figure S3L) than CAR^+^CD4^+^ G28zT2 T cells. Collectively, these results demonstrate that the T28zT2 CAR molecule prevents T cell exhaustion in tumors.

### CD4^+^ but not CD8^+^ T28zT2 T cells are effective for tumor growth inhibition

As CD4^+^ but not CD8^+^ T28zT2 T cells persisted in murine peripheral blood and xenografted tumors (Figures 1J and 1M), we assessed whether the CD4^+^ or CD8^+^ compartment contributed to antitumor effects of T28zT2 T cells. It was easier for CD4^+^ T28zT2 T cells than CD8^+^ T28zT2 T cells to be fully activated by soluble TGF-β1 (Figure S4A). CD4^+^ T28zT2 T cells and CD4^+^ G28zT2 T cells both lysed Huh7 cells, compared with CD4^+^ 1928zT2 T cells (Figure S4B). Conversely, CD8^+^ T28zT2 T cells did not lyse Huh7 cells as efficiently as CD8^+^ G28zT2 T cells or exhibited superior lysing capacity compared to CD8^+^ 1928zT2 T cells (Figure S4C). CD4^+^ T28zT2 T cells secreted more Granzyme B, IFN-γ, perforin (Figures S4D–S4F), and IL-2 than CD4^+^ 1928zT2 T cells (Figure S4G), while CD8^+^ T28zT2 T cells produced minimal amounts of Granzyme B and IFN-γ, compared to CD8^+^ G28zT2 T cells (Figures S4H and S4I).

**Figure 3:**
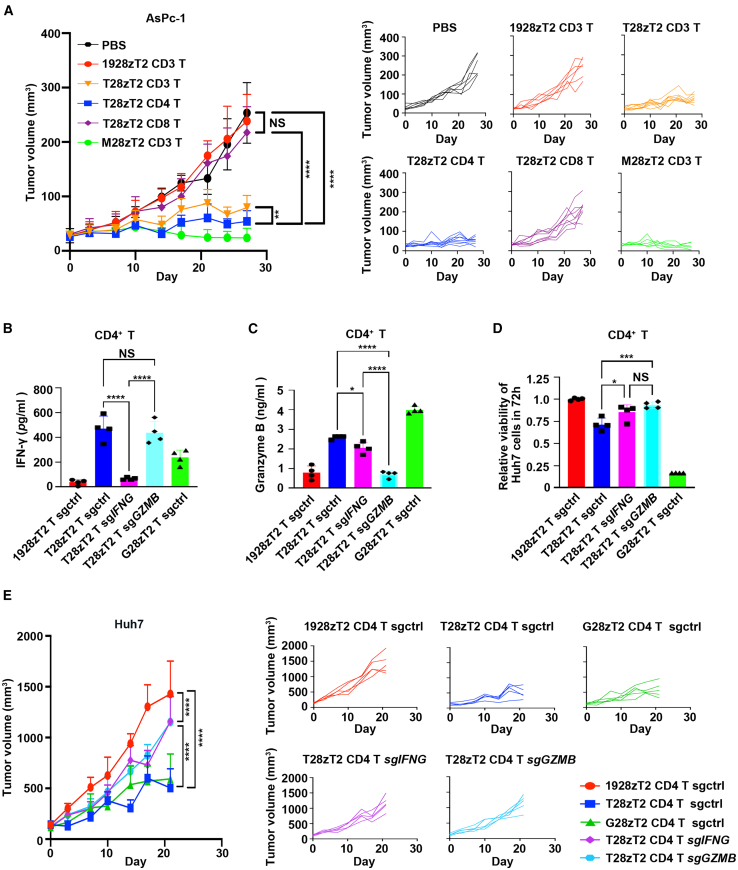
CD4^+^ but not CD8^+^ T28zT2 T cells are effective for tumor growth inhibition (A) CD4^+^ and CD8^+^ T cell compartments were sorted, and CD8^+^ T28zT2 T cells and CD4^+^ T28zT2 cells were generated. We also mixed the CD4^+^ and CD8^+^ T28zT2 T cells at a ratio of 1:1 to obtain CD3^+^ T28zT2 T cells. We then infused a total of 5 × 10^6^ of the three types of T28zT2 T cells, CD3^+^ 1928zT2 T cells, or CD3^+^ M28zT2 T cells peritumorally into subcutaneous xenografts of NSI mice inoculated with 1 × 10^6^ AsPc-1 cells (day 0). Tumor volumes were monitored on the indicated days (*n* = 8 mice/T28zT2 CD3, T28zT2 CD4, and T28zT2 CD8 groups; *n* = 6 mice/1928zT2 CD3, M28zT2 CD3 and PBS groups); data are the mean ± SD values; two-way ANOVA with Tukey’s multiple comparisons test; ∗∗*p*≤ 0.01, ∗∗∗∗*p*≤ 0.0001. (B–D) CRISPR-Cas9-RNP knockout of *GZMB* or *IFNG* expression in CD4^+^ T28zT2 T cells. A total of 4 × 10^5^ T28zT2 CD4 control single guide RNA (sgctrl), T28zT2 CD4 single guide RNA targeting granzyme B (sg*GZMB*), T28zT2 CD4 single guide RNA targeting interferon-gamma (sg*IFNG*), G28zT2 CD4 sgctrl, or 1928zT2 CD4 sgctrl T cells were cocultured with 1 × 10^5^ Huh7 cells for 72 h. Shown are the levels of IFN-γ (B) and Granzyme B (C) (from 4 independent experiments) detected by ELISA assay; (D) the relative viability of Huh7 cells (from 4 independent experiments); data are the mean ± SEM values; one-way ANOVA with Tukey’s multiple comparisons test; ∗*p* < 0.05, ∗∗∗*p*≤ 0.001, ∗∗∗∗*p*≤ 0.0001. (E) Curves showing variations in the volume of Huh7 tumors in NSI mice post-infusion of T28zT2 CD4 sgctrl, T28zT2 CD4 sg*IFNG*, T28zT2 CD4 sg*GZMB*, G28zT2 CD4 sgctrl, or 1928zT2 CD4 sgctrl T cells (*n* = 5 mice/group); data are the mean ± SD values; two-way ANOVA with Tukey’s multiple comparisons test; ∗∗∗∗*p*≤ 0.0001. See also in Figures S4 and S5.

In xenografts, CD3^+^ T28zT2 T cells, CD4^+^ T28zT2 T cells, and CD3^+^ M28zT2 T cells significantly inhibited the growth of AsPc-1, a pancreatic cancer cell line characterized by high TGF-β1 and MSLN expression (Figures S4J and S4K), compared to CD8^+^ T28zT2 T cells, CD3^+^ 1928zT2 T, cells or PBS treatment, none of which exhibited antitumor effects (Figure 3A). Moreover, we found that tumor-infiltrating CAR^+^CD4^+^ T28zT2 cells but not CAR^+^CD4^+^ 1928zT2 cells upregulated NKG2D expression (Figures S4L–S4O). Additionally, AsPc-1 cells expressed NKG2D ligands such as MICA/B and ULBP2/5/6 (Figures S4P and S4Q), suggesting that CD4^+^ T28zT2 cells recognized AsPc-1 cells via NKG2D within tumors.

The viability of Huh7 cells in cocultures with CD4^+^ T28zT2 T cells with Granzyme B or IFN-γ ablation (Figures 3B and 3C) was significantly increased upon ablation of Granzyme B or IFN-γ (Figure 3D). Moreover, *in vivo*, the antitumor effects of sg*GZMB*-transduced and sg*IFNG*-transduced CD4^+^ T28zT2 T cells were compromised in Huh7 xenografts, compared to sgctrl-transduced CD4^+^ T28zT2 T cells (Figure 3E). Taken together, these results suggest that CD4^+^ T28zT2 T cells eliminated cancer cells mainly through secreting Granzyme B and IFN-γ.

### Anti-TGF-β CAR T cells did not cause toxicity *in vivo*

We next evaluated the safety of T28zT2 T cells *in vivo* by injection of a high number of T28zT2 T cells into tumor-free NSI mice. T28zT2 T cells did not cause any damage to the lung, liver, and kidneys of NSI mice on day 7 or 14 after infusion, similar to 1928zT2 T cells (Figures S5A–S5C).

We also assessed the toxicity of autologous anti-TGF-β CAR T cells in immunocompetent C57BL/6 mice. Increase of *in vitro* CD69 expression and IL-2 production in T28zT2-transduced Jurkat cells after murine TGF-β1 treatment (Figures S5D and S5E) suggested that murine TGF-β1 could activate T28zT2. Therefore, we generated a murine version of the anti-TGF-β CAR vector (mu-T28zT2), containing an anti-TGF-β scFv, the murine CD28 transmembrane and intracellular domains, the murine CD3ζ signaling domain, and the murine TLR2 domain (Figures S5F and S5G). A murine version of the anti-murine CD19 CAR vector (mu-m1928zT2) was used as a control (Figures S5F and S5G). C57BL/6 mice were transferred with either mu-T28zT2 or mu-m1928zT2 T cells on day 0 and monitored for 12 weeks. All the mice from both groups survived and did not exhibit any adverse effects (Figure S5H) or T cell infiltration into any examined tissues (Figure S5I).

We further evaluated the toxicity of T28zT2 T cells in AsPc-1 xenografts by infusing T28zT2 T cells or 1928zT2 T cells at a high dose on day 0 and examining the morphology of their paracancerous tissues, livers, and kidneys at multiple time points (Figure S5J). T28zT2 T cells impeded AsPc-1 growth (Figure S5K) and prolonged the survival of xenografts, compared to 1928zT2 T cells (Figure S5L). Of note, no T cell toxicity was detected in the collected tissues from the T28zT2 group (Figures S5M–S5R). Taken together, these results indicate that T28zT2 T cells did not result in severe toxicity *in vivo*.

### A combination of CD4^+^ anti-TGF-β CAR T cells and CD8^+^ anti-GPC3 or anti-MSLN CAR T cells exhibits augmented antitumor effects

**Figure 4:**
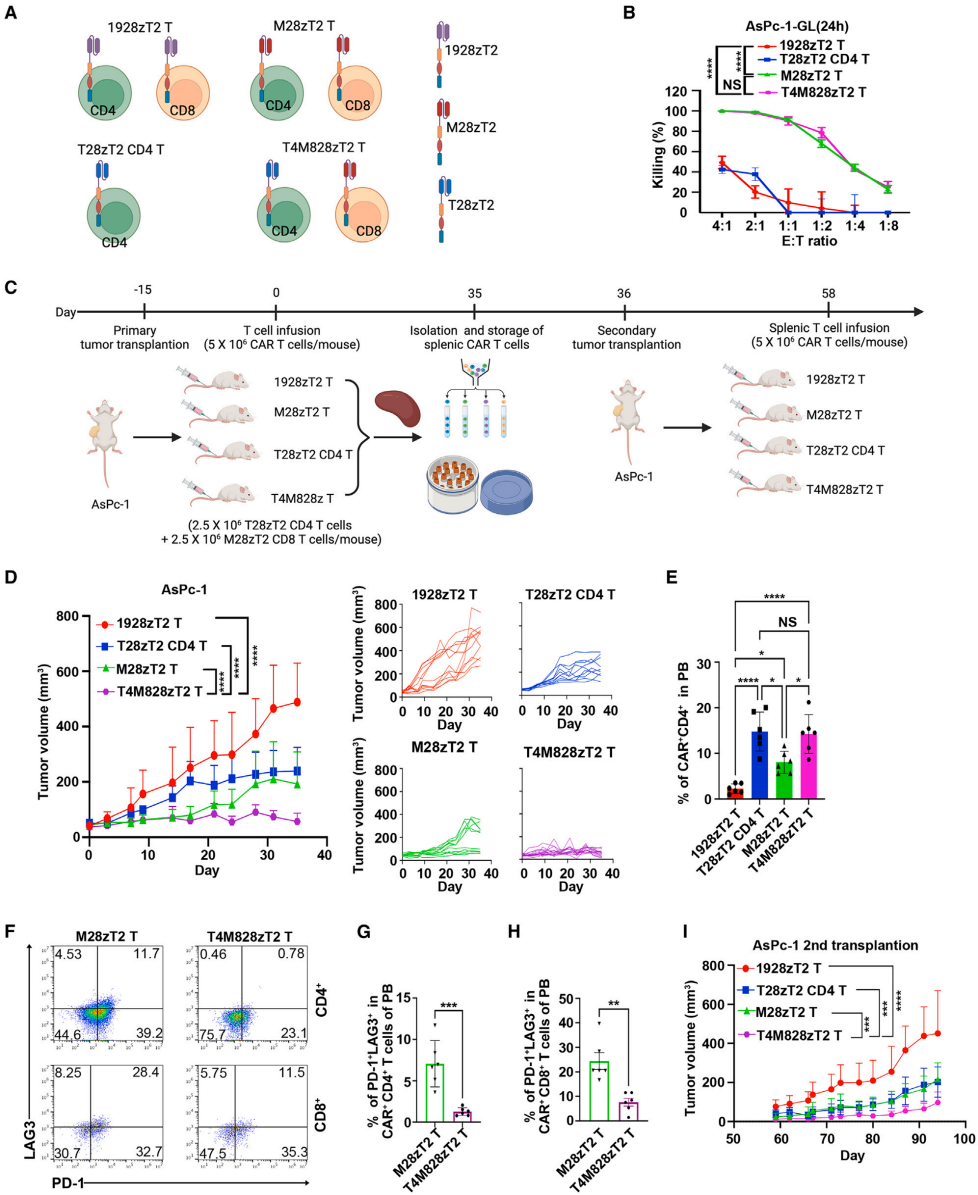
A combination of CD4^+^ anti-TGF-β CAR T cells and CD8^+^ anti-MSLN CAR T cells exhibits augmented antitumor effects in AsPc-1 tumor models (A) Mixed CAR T cells consisted of CD4^+^ T28zT2 T cells and CD8^+^ M28zT2 T cells, designated T4M828zT2 T cells. Graphics were created with BioRender.com (agreement number *VP27TZMIXE*). (B) The percentage of AsPc-1-GL cells with 1928zT2, M28zT2, T28zT2 CD4, or T4M828zT2 T cell-induced lysis overnight; data are the mean percentage of tumor cell-specific lysis ± SEM values; *n* = 3 independent experiments; two-way ANOVA with Tukey’s multiple comparisons test; ∗∗∗∗*p* ≤ 0.0001. (C) A schematic diagram of the experimental design. 8-week-old male NSI mice were inoculated subcutaneously with 2 × 10^6^ AsPc-1 tumor cells into the right flank. A total of 5 × 10^6^ T4M828zT2 T, M28zT2 T, and 1928zT2 T cells were injected peritumorally (day 0). At each endpoint, the splenic CAR T cells were sorted by fluorescence-activated cell sorting (FACS) and cryopreserved. Graphics were created with BioRender.com (agreement number *GX26UUJLAJ*). (D) Tumor volumes were monitored on the indicated days (*n* = 11 mice/1928zT2 and M28zT2 group, *n* = 10 mice/T28zT2 CD4 group, *n* = 15 mice/T4M828zT2 group). The majority of mice in all groups exhibited serious symptoms of GVHD, halting animal experiments on day 35 post-injection. Data are shown for 2 independent experiments; displayed as the mean ± SEM values; a repeat measures ANOVA with Tukey’s multiple comparisons test; ∗∗∗∗*p*≤ 0.0001 (T4M28zT2 T cells vs. 1928zT2 T cells on day 35, T4M28zT2 T cells vs. M28zT2 T cells on day 35, and T4M28zT2 T cells vsT28zT2 CD4 T cells on day 35). (E) The percentages of CAR^+^(GFP^+^) CD4^+^ T cells in murine peripheral blood (PB) populations of mice from the T4M828zT2 and M28zT2 groups on day 35 were determined by flow cytometry. *n* = 6 mice/group; data are the mean ± SD values; one-way ANOVA with Tukey’s multiple comparisons test; ∗*p* < 0.05; ∗∗∗∗*p* ≤ 0.0001. (F–H) The percentages of PD-1^+^LAG3^+^ expression among CAR^+^CD4^+^(F and G) and CAR^+^CD8^+^ (F and H) T cells in murine peripheral blood (PB) of mice from the T4M828zT2 and M28zT2 groups on day 35 were determined by flow cytometry. *n* = 6 mice/group; (G and H) data are the mean ± SD values; unpaired two-tailed t test; ∗∗*p*≤ 0.01, ∗∗∗*p*≤ 0.001. (I) 8-week-old male NSI mice were inoculated subcutaneously with 2 × 10^6^ AsPc-1 tumor cells into the right flank. A total of 5 × 10^6^ T4M828zT2 T, T28zT2 CD4, M28zT2 T, or 1928zT2 splenic T cells were injected peritumorally. Tumor volumes were monitored on the indicated days (*n* = 5 mice/group); data are shown as the mean ± SD values; ∗∗∗*p*≤ 0.001, ∗∗∗∗*p*≤ 0.0001. See also in Figure S6.

As CD4^+^ rather than CD8^+^ compartments of T28zT2 T cells exhibited antitumor activity, we evaluated the efficacy of a combination of CD4^+^ T28zT2 T cells and CD8^+^ conventional CAR T cells, such as M28zT2 or G28zT2 T cells (Figure 4A). The combination of CD4^+^ T28zT2 T cells and CD8^+^ M28zT2 T cells (T4M828zT2) eliminated AsPc-1 cells as potently as a conventional mixture of CD4^+^ and CD8^+^ M28zT2 T cells, compared to a conventional mixture of CD4^+^ and CD8^+^ 1928zT2 T cells (Figure 4B). Strikingly, in xenografts, T4M828zT2 T cells were more effective than CD3^+^ M28zT2 T cells or CD4^+^ T28zT2 T cells alone for suppressing AsPc-1 growth (Figures 4C and 4D). Moreover, the percentages of CAR^+^CD4^+^ T cells in murine peripheral blood from the CD4^+^ T28zT2 and T4M828zT2 group were significantly higher than those from the M28zT2 or 1928zT2 group (Figure 4E). Additionally, the frequencies of exhausted T cells (PD-1^+^LAG3^+^) in the CD4^+^CAR^+^ or CD8^+^CAR^+^ compartments from the T4M828zT2 group were significantly lower than those from the M28zT2 group (Figures 4F–4H). To further assess the persistence of these CAR T cells (Figure 4C), we isolated them from the spleen of AsPc-1 xenografts animals and infused them into another group of AsPc-1 xenografts. Secondary transplanted T4M28zT2 T cells inhibited tumor growth better than CD3^+^ M28zT2, CD4^+^ T28zT2, or CD3^+^ 1928zT2 T cells alone (Figure 4I).

We next assessed the efficacy of this combination of CD4^+^ T28zT2 T cells and CD8^+^ G28zT2 T cells (T4G828zT2) in Huh7 xenografts. Huh7 cells grew slower in the T4G828zT2 group, compared to the groups that were infused with conventional CD3^+^ G28zT2 T cells or CD3^+^ 1928zT2 T cells (Figure S6A). Furthermore, the frequencies of exhausted T cells (PD-1^+^LAG3^+^) in the CD4^+^CAR^+^ and CD8^+^CAR^+^ compartments from the T4G828zT2 group were significantly lower than those from the G28zT2 group (Figures S6B–S6D).

**Figure 5:**
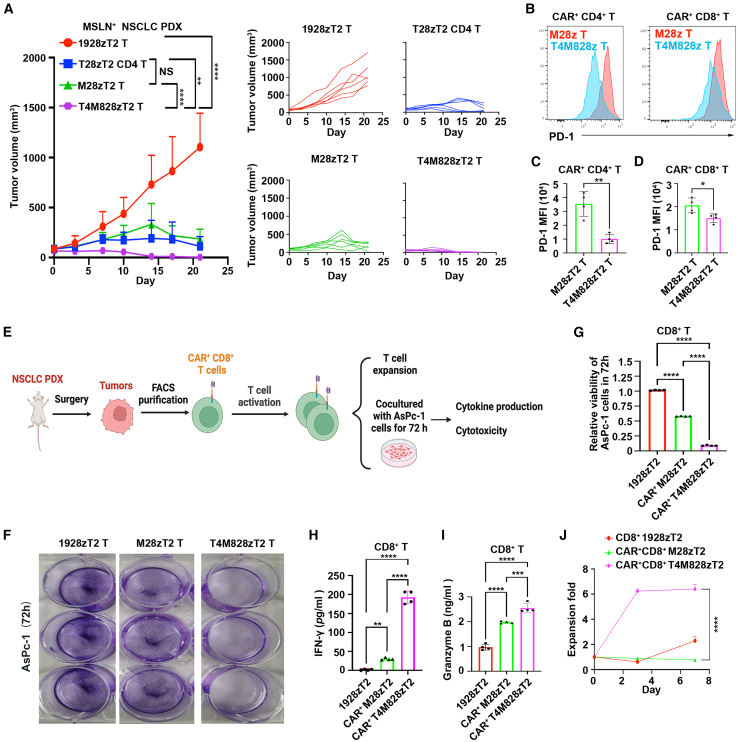
A combination of CD4^+^ anti-TGF-β CAR T cells and CD8^+^ anti-MSLN CAR T cells exhibits augmented antitumor effects in NSCLC PDX (A) NSCLC PDX tumors were diced into ∼30 mm^3^ pieces, and tissue were inoculated subcutaneously into the right flanks of 8-week-old male NSI mice. 5 × 10^6^ T4M828zT2, T28zT2 CD4, M28zT2, or 1928zT2 T cells were injected peritumorally (day 0). Tumor volumes were monitored on the indicated days (*n* = 6 mice/group); data are the mean ± SD values; two-way ANOVA with Tukey’s multiple comparisons test; ∗∗*p*≤ 0.01, ∗∗∗∗*p*≤ 0.0001. (B–D) The mean fluorescence intensity (MFI) of PD-1 among tumor-infiltrating CAR^+^CD4^+^ (B, left) and CAR^+^CD8^+^ (B, right) T cells from the T4M828zT2 and M28zT2 groups on day 21 determined by flow cytometry (*n* = 4 mice/group). (C and D) Data are shown as the mean ± SD values; unpaired two-tailed t test; ∗*p* < 0.05, ∗∗*p*≤ 0.01. (E) A schematic diagram of the experimental design. Tumor tissue from 1928zT2, M28zT2, or T4M828zT2 groups was obtained from NSCLC PDX models at the endpoint (day 21). Tumor tissues were prepared into single-cell suspension. Tumor-infiltrating CAR^+^(GFP^+^) CD8^+^ T cells from the M28zT2 and T4M828zT2 groups and tumor-infiltrating CD8^+^ T cells from the 1928zT2 group were sorted by FACS. These tumor-infiltrating T cells were then stimulated with CD3/CD28 monoclonal antibodies (mAbs) and subjected to functional experiments. Finally, the cytotoxicity, cytokine production, and T cell expansion of tumor-infiltrating CD8^+^ T cells from the 1928zT2, M28zT2, and T4M828zT2 groups were evaluated. Graphics were created with BioRender.com (agreement number *QW27PPO9DF*). (F–I) Tumor-infiltrating CD8^+^ 1928zT2, CAR^+^CD8^+^ M28zT2, or CAR^+^CD8^+^ T4M828zT2 cells were incubated with AsPc-1 cells at a 2:1 effector (E):target (T) ratio for 72 h. (F) Representative images of 0.1% crystal violet staining of AsPc-1 cells cocultured with CD8^+^ 1928zT2, CAR^+^CD8^+^ M28zT2, or CAR^+^CD8^+^ T4M828zT2 tumor-infiltrating T cells *ex vitro*. (G) The relative viability of AsPc-1 cells with 1928zT2, M28zT2, or T4M828zT2 T-cell induced lysis after 72 h *n* = 4 mice/group. (H and I) Supernatants were harvested and analyzed with a multiplex immunoassay to determine the concentrations of the indicated cytokines. *n* = 4 mice/group. The concentrations of IFN-γ (H) and Granzyme B (I) were measured by ELISA assay; data are the mean ± SD values; one-way ANOVA with Tukey’s multiple comparisons test; ∗∗∗*p* ≤ 0.001, ∗∗∗∗*p* ≤ 0.0001. (J) The expansion of tumor-infiltrating CD8^+^ 1928zT2, CAR^+^CD8^+^ M28zT2, and CAR^+^CD8^+^ T4M828zT2 cells was detected by flow cytometry on day 0, 3 and 7; data are the mean ± SD values; *n* = 4 mice/group; two-way ANOVA with Tukey’s multiple comparisons test; ∗∗∗∗*p* ≤ 0.0001. See also in Figure S6.

Finally, we evaluated the efficacy of T4M828zT2 T cells in non-small cell lung cancer (NSCLC) PDX models.^46^ T4M828zT2 T cells were also more effective than CD3^+^ M28zT2 T cells or CD4^+^ T28zT2 T cells alone at suppressing the growth of NSCLC primary tumors (Figure 5A). Additionally, PD-1 expression in tumor-infiltrating CAR^+^CD4^+^ or CAR^+^CD8^+^ T cells in the T4M828zT2 group was lower than that in the M28zT2 group (Figures 5B–5D). We then purified CAR^+^CD8^+^ T cells from tumors of these NSCLC PDX models and performed functional assays upon TCR activation (Figure 5E). Tumor-infiltrating CAR^+^CD8^+^ T cells from the T4M828zT2 group lysed AsPc-1 cells more efficiently (Figures 5F and 5G), produced more IFN-γ and Granzyme B (Figures 5H and 5I), and expanded faster (Figure 5J) than those from the M28zT2 or 1928zT2 groups. We also assessed the efficacy of T4G828zT2 T cells in an HCC PDX model and found thar the tumor sizes of the T4G828zT2 group were smaller than those of mice that were infused with CD3^+^ G28zT2 T cells or CD4^+^ T28zT2 T cells (Figure S6E). Taken together, these results demonstrate that the combination of CD4^+^ T28zT2 T cells and CD8^+^ CAR T cells was a more effective antitumor treatment than the conventional mixture of CD4^+^ and CD8^+^ CAR T cells.

### CD4^+^ anti-TGF-β CAR T cells maintained mitochondrial fusion upon TGF-β1 treatment

**Figure 6:**
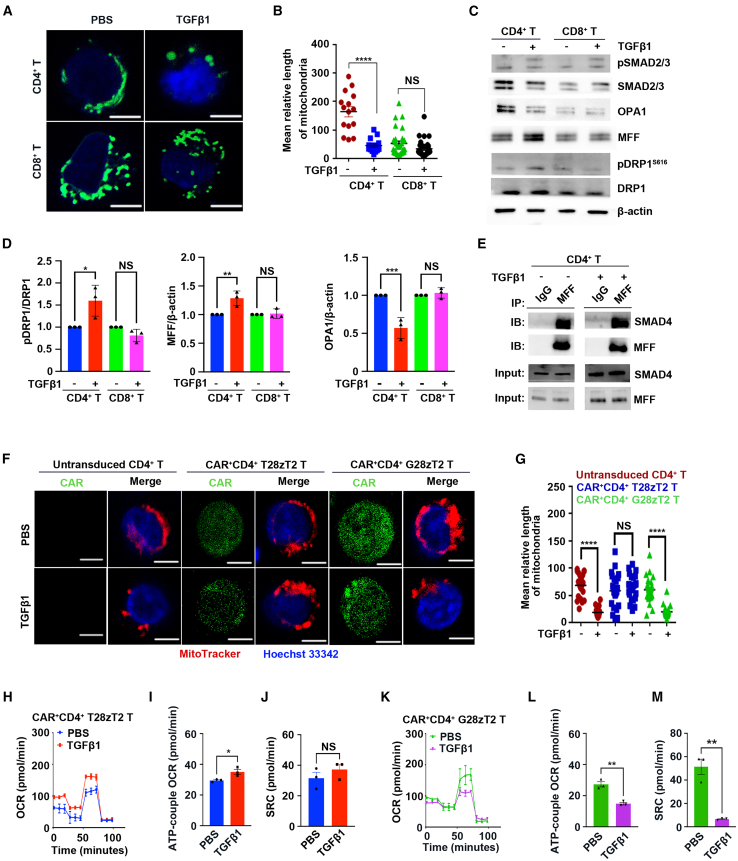
TGF-β1 suppressed OXPHOS activity in CD4^+^ human T cells (A–D) CD4^+^ and CD8^+^ T cells (1 × 10^6^) were activated with CD3/CD28 mAbs for 24 h, followed by PBS or TGF-β1 (10 ng/mL) treatment for 16 h. (A) Mitochondrial morphology of CD4^+^ and CD8^+^ T cells upon PBS or TGF-β1 (10 ng/mL) treatment, as determined by spinning disk confocal microscopy. Mitochondria are green (MitoTracker Green), and nuclei are blue (DAPI). Scale bar, 5 μm. (B) Relative lengths of mitochondria, as analyzed by ImageJ software, in CD4^+^ and CD8^+^ T cells (2 independent experiments). Each dot represents the mean relative length of the mitochondria in a sample. Data are shown as the mean ± SEM values; paired two-tailed t test; ∗∗∗∗*p*≤ 0.0001. (C) Immunoblot analysis of cellular protein extracts probed with antibodies against pSMAD2^S465/467^ (top)/pSMAD3^S423/425^ (bottom) (pSMAD2/3), SMAD2/3, OPA1, MFF, pDRP1^S616^, DRP1, and β-actin. (D) The relative expression of pDRP1^S616^, MFF, and OPA1 was analyzed by ImageJ software (3 independent experiments). Data are shown as the mean ± SEM values; paired two-tailed t test; ∗*p* < 0.05, ∗∗*p*≤ 0.01, ∗∗∗*p*≤ 0.001. (E) Immunoprecipitation (IP) of MFF in activated CD4^+^ T cells after treatment with PBS or TGF-β1 (10 ng/mL) for 2 h and subsequent immunoblot (IB) analysis of the indicated proteins. (F) Mitochondrial morphology of CAR^+^CD4^+^ T28zT2, CAR^+^CD4^+^ G28zT2, and untransduced CD4^+^ T cells upon treatment with PBS or TGF-β1 (10 ng/mL) as determined by spinning disk confocal microcopy. Mitochondria are red (MitoTracker Deep Red), CAR T cells are green (GFP), and nuclei are blue (Hoechst 33342). Scale bar, 5 μm. (G) Lengths of mitochondria, as analyzed by ImageJ software (2 independent experiment), in CAR^+^CD4^+^ T28zT2, CAR^+^CD4^+^ G28zT2, and untransduced CD4^+^ T cells. Each dot represents the mean relative length of the mitochondria in a sample. (H–M) OCR profile (H and K), ATP-coupled OCR (I and L), and SRC (J and M) of CAR^+^CD4^+^ T28zT2 T cells and CAR^+^CD4^+^ G28zT2 T cells (3 independent experiments). Data are shown as the mean ± SEM values; unpaired two-tailed t test; ∗*p* < 0.05, ∗∗*p*≤ 0.01. See also in Figure S6.

T cell fate is affected by their metabolism and mitochondrial dynamics,^14^^,^^47^ which are regulated by OPA1, MFN1/2, DRP1, and mitochondrial fission factor (MFF).^10^^,^^48^^,^^49^^,^^50^ We treated purified CD4^+^ and CD8^+^ single-positive T cells with TGF-β1 and observed that, while TGF-β1-treated CD4^+^ T cells exhibited predominantly punctate mitochondria, mitochondria in untreated CD4^+^ T cells formed elongated tubules (Figure 6A). This significant mitochondrial length reduction in TGF-β1-treated cells was specific to CD4^+^ T cells and not observed in CD8^+^ T cells (Figures 6A and 6B). TGF-β1 also reduced the mitochondrial membrane potential evaluated by tetramethylrhodamine methyl ester (TMRM) staining in CD4^+^ T cells, but not in CD8^+^ T cells (Figure S6F). Additionally, TGF-β1 reduced the baseline oxygen consumption rate (OCR) (Figures S6G annd S6H), the adenosine triphosphate (ATP)-coupled OCR (Figure S6I), and spare respiratory capacity (SRC) (Figure S6J) in CD4^+^ T cells; showing that TGF-β1 decreased the respiratory capacity of CD4^+^ T cells. Conversely, TGF-β1 did not affect the basal OCR, ATP-coupled OCR, or SRC in CD8^+^ T cells (Figures S6K–S6N).

We then measured the protein levels of pSMAD2/3, OPA1, MFF, DRP1, and pDRP1^S616^ in TGF-β1-treated CD4^+^ and CD8^+^ T cells and found that the phosphorylation of SMAD2 was augmented upon TGF-β1 treatment (Figure 6C). In TGF-β1-treated CD4^+^ T cells, OPA1 expression was decreased while the MFF and pDRP1^S616^ protein levels were increased (Figures 6C and 6D). However, interestingly, after TGF-β1 treatment of CD8^+^ T cells, the levels of OPA1, MFF, or pDRP1^S616^ were not altered (Figures 6C and 6D). Surprisingly, co-immunoprecipitation assays revealed that MFF physically interacted with SMAD4 in CD4^+^ T cells (Figure 6E). In contrast, TCR activation rather than TGF-β treatment induces SMAD4 translocation in CD8^+^ T cells.^51^ These differences may contribute to the distinct effects of TGF-β on CD4^+^ and CD8^+^ T cells.

To examine whether T28zT2 molecules can prevent this TGF-β1-induced mitochondrial fission in human CD4^+^ T cells, we cocultured T28zT2 CD4^+^ T cells or G28zT2 CD4^+^ T cells with Huh7 cells in the presence of TGF-β1. Confocal micrographs revealed that the mitochondrial length did not change in CAR^+^CD4^+^ T28zT2 T cells cocultured with Huh7 cells in the presence of TGF-β1 (Figures 6F and 6G). In contrast, the mitochondrial length was decreased significantly in untransduced CD4^+^ T cells and in CAR^+^CD4^+^ G28zT2 cells under the same conditions (Figures 6F and 6G). Upon TGF-β1 activation, the ATP-coupled OCR was elevated and the SRC remained unchanged in CAR^+^CD4^+^ T28zT2 T cells (Figures 6H–6J), whereas the ATP-coupled OCR and SRC were decreased in CAR^+^CD4^+^ G28zT2 T cells (Figures 6K–6M). Therefore, T28zT2 molecules prevented TGF-β1-induced mitochondrial fission in CD4^+^ T cells.

## Discussion

In this study, we demonstrated that anti-TGF-β CAR T cells exhibit antitumor effects in xenografts of multiple cancers. Particularly, CD4^+^ but not CD8^+^ T28zT2 T cells expanded robustly and inhibited tumor growth *in vivo*. This observation is in line with previous studies that the inhibition of tumor growth caused by TGF-βRII-deficient T cells is dependent on Th2 immunity involving murine CD4^+^ T cells, rather than murine CD8^+^ T cells.^52^^,^^53^ The different dynamics and efficacies of the CD4^+^ and CD8^+^ compartments of T28zT2 T cells are possibly due to distinct sensitivity to TGF-β1 in these cells. TGF-β1 efficiently suppresses type 1 and type 2 immunity and IFN-γ production in CD4^+^ T cells,^52^^,^^54^ while CD8^+^ T cell function modulation is more dependent on TCR signaling than TGF-β signaling.^51^ Mechanistically, our findings show that TGF-β1 specifically induced mitochondrial fission and suppressed the mitochondria potential in CD4^+^ T cells, but not in CD8^+^ T cells.

Tumor-infiltrating CD4^+^ T28zT2 T cells exhibited phenotypes of memory-like T cells with high CXCR3 expression. Recent studies show that CXCR3, repressed by TGF-β1,^43^ is positively correlated with improved intratumoral T cell infiltration^44^ and good prognosis in anti-PD-1 therapies.^45^ CD4^+^ T28zT2 T cells upregulated NKG2D within tumors and suppressed tumor growth by producing IFN-γ and Granzyme B. Previous studies consistently report that TGF-β1 suppresses the expression of NKG2D in T cells and NK cells.^55^^,^^56^^,^^57^ It is possible that CD4^+^ T28zT2 T cells recognized cancer cells via NKG2D, formed immunological synapses, and delivered IFN-γ and Granzyme B specifically to cancer cells through the immunological synapses. This may explain why CD4^+^ T28zT2 T cells did not cause severe toxicity in tumor-free mice and xenografts.

The combination of CD4^+^ anti-TGF-β CAR T cells and conventional CD8^+^ CAR T cells exhibited synergistic and augmented antitumor effects. We hypothesize this is because CD4^+^ anti-TGF-β CAR T cells lysed cancer cells, prevented exhaustion of CD8^+^ conventional CAR T cells, and improved the CD8^+^ conventional CAR T cells’ efficacy. Consistently, CD4^+^ T cells enhance the effector function of CD8^+^ T cells by downregulating their expression of coinhibitory receptors.^58^ Based on our preclinical findings, we initiated a phase 1 clinical investigation to assess the antitumor efficacy of T4G828zT2 or T4M828zT2 T cells for treating relapsed and refractory HCC or pancreatic cancer (ClinicalTrials.gov: NCT03198052).

In conclusion, our findings indicate that rewiring the TGF-β signaling pathway of CD4^+^ T cells using an anti-TGF-β CAR led to anticancer immunity of anti-GPC3 CAR or anti-MSLN CAR CD8 T cells, rendered T cells resistant to exhaustion, and thus provides a strategy for TGF-β1^+^GPC3^+^ or TGF-β1^+^MSLN^+^ solid tumor patients.

### Limitations of the study

The hypothesis that the lack of toxicity is due to T28zT2 T cells forming an immunological synapse with tumor cells via NKG2D is indicated on the observation that CD4^+^ T28zT2 T cells upregulated NKG2D in tumors and is speculative. The existing data have not fully elucidated why CD4^+^ T28zT2 T cells are able to achieve such robust efficacy without any toxicity given the proposed mechanism of action, which is non-antigen specific. Further experiments are warranted to evaluate the hypothesis in the future.

## Resource availability

### Lead contact

Further information and requests for resources should be directed to and will be fulfilled by the lead contact, Peng Li (li_peng@gibh.ac.cn).

### Materials availability

This study did not generate new unique reagents.

### Data and code availability

• The original data of the bulk RNA-seq have been deposited at the Genome Sequence Archive for Human (GSA for Human) (https://ngdc.cncb.ac.cn/gsa-human/browse/HRA001397) and are publicly available as of the date of publication. Accession number (HRA001397) is listed in the key resources table.
• Original western blot data have been deposited at Mendeley data (https://data.mendeley.com/drafts/n7htzcxmw4/1) and are publicly available as of the date of publication. Accession number (https://doi.org/10.17632/n7htzcxmw4.1) is listed in the key resources table. Microscopy data reported in this paper will be shared by the lead contact upon request.
• This paper does not report original code.
• Any additional information required to reanalyze the data reported in this paper is available from the lead contact upon request.

## Acknowledgments

This study was equally supported by the 10.13039/501100001809National Natural Science Foundation of China, no. 82341204 (P.L.); the Strategic Priority Research Program of the Chinese Academy of Sciences, no. XDB0940301 (P.L.); and National Key Research and Development Plan, no. 2022YFE0210600 (S.L.). The study was also supported by the International Partnership Program of The Chinese Academy of Sciences, no. 188GJHZ2022015GC (P.L.); the 10.13039/501100001809National Natural Science Foundation of China, 82202031 (L.Q.), 32370996 (L.Q.), 82273377 (S.L.), 82402150 (Dr. Yuanbin Cui), and 32170946 (Dr. Zhiwu Jiang); 10.13039/501100012245Science and Technology Planning Project of Guangdong Province, no. 2024A0505040021 (P.L.), 2023B1212060050, and 2023B1212120009; Science and Technology Projects in Guangzhou, China, no. 2024B03J1232 (P.L.); Science and Technology Program of Guangzhou No.2023A04J0415 (Dr. Di Wu); 10.13039/501100021171Guangdong Basic and Applied Basic Research Foundation, no. 2022A1515110349 (D.Z.), 2021A1515110005 (L.Q.), 2022A1515012484 (S.L.), 2022A1515012569 (Dr. Zhiwu Jiang), 2022A1515012360 (L.Q.), 2022A1515010604 (Y.Y.), and 2024B1515040020 (Professor Hui Zheng); Guangdong-Hong Kong-Macau Joint Laboratory of Respiratory Infectious Diseases, no. 2019B121205010 (P.L.); Basic Research Project of Guangzhou Institutes of Biomedicine and Health, 10.13039/501100002367Chinese Academy of Sciences, no. GIBHBRP23-03 (P.L.); the University Grants Committee/Research Grants Council of the Hong Kong Special Administrative Region, China (Project No. AoE/M-401/20), Innovation  and  Technology Fund (ITF); Guangdong Province Grant for Belt and Road Joint Laboratory, no. 2022B1212050004; and the Youth Innovation Promotion Association of the Chinese Academy of Sciences, no. 2020351 (Dr. Zhiwu Jiang) and 2023371 (S.L.). The graphical abstract and schematic diagrams were created using BioRender.com.

## Author contributions

P.L., D.Z., and L.Q. conceived and designed the research; D.Z., L.Q., J. Lv, M.C., Y.Z., and M.L. performed *in vitro* assays and animal experiments; L.Q., D.Z., and J.Lv. optimized the protocol for manufacturing CAR T cells; P.L., D.Z., and L.Q. wrote the manuscript; B.H., S.L., Y.Q., Z.T., B.-C.W., Y.-L.W., R.W., G.C., Y.Y., N.W., J. Lau, J.P.T., B.P., D.Q., K.X., and Z.Z. provided critical advices on this study and revised the manuscript; K.X. and Z.Z. provided important research reagents and technical advices; and all authors approved the manuscript.

## Declaration of interests

P.L. and Z.T. are founders of GZCBL and have equity in GZCBL. There is a pending patent related to this research work.

## STAR★Methods

### Key resources table

**Table:** 

REAGENT or RESOURCE	SOURCE	IDENTIFIER
**Antibodies**		

Purified anti-human CD45RA (Maxpar® Ready) Antibody	Biolegend	Cat# 304143 RRID: AB_2562822
Purified anti-human CD45RO (Maxpar® Ready) Antibody	Biolegend	Cat# 304239 RRID: AB_2563752
Purified anti-human CD3 (Maxpar® Ready) Antibody	Biolegend	Cat# 300443 RRID: AB_2562808
Purified anti-human CD8 (Maxpar® Ready) Antibody	Biolegend	Cat# 344727 RRID: AB_2563762
Purified anti-human CD4 (Maxpar® Ready) Antibody	Biolegend	Cat# 300541 RRID: AB_2562809
Purified anti-human CD197 (CCR7) (Maxpar® Ready) Antibody	Biolegend	Cat# 353237 RRID: AB_2563726
Purified anti-human CD56 (NCAM) (Maxpar® Ready) Antibody	Biolegend	Cat# 318345 RRID: AB_2562830
Purified anti-human CX3CR1 Antibody	Biolegend	Cat# 341602 RRID: AB_1595422
Purified anti-human CD314 (NKG2D) Antibody	Biolegend	Cat# 320802 RRID: AB_492956
Purified anti-human CD27 (Maxpar® Ready) Antibody	Biolegend	Cat# 302839 RRID: AB_2562817
Purified anti-human CD28 (Maxpar® Ready) Antibody	Biolegend	Cat# 302937 RRID: AB_2563737
Purified anti-human CD69 (Maxpar® Ready) Antibody	Biolegend	Cat# 3130939 RRID: AB_2562827
Purified anti-human TIM-3 (Maxpar® Ready) Antibody	Biolegend	Cat# 345019 RRID: AB_2563790
Purified anti-human PD-1 (Maxpar® Ready) Antibody	Biolegend	Cat# 329941 RRID: AB_2563734
Purified anti-human CD152 (CTLA-4) (Maxpar® Ready) Antibody	Biolegend	Cat# 369602 RRID: AB_2566610
Purified anti-human CD197 (CCR7) (Maxpar® Ready) Antibody	Biolegend	Cat# 353237 RRID: AB_2563726
Purified anti-human CD62L (Maxpar® Ready) Antibody	Biolegend	Cat# 304835 RRID: AB_2563758
Purified anti-human CD127(IL7R) (Maxpar® Ready) Antibody	Biolegend	Cat# 351337 RRID: AB_2563715
Purified anti-human TCF1 (Maxpar® Ready) Antibody	Biolegend	Cat# 655202 RRID: AB_2562103
Purified anti-human T-bet (Maxpar® Ready) Antibody	Biolegend	Cat# 644825 RRID: AB_2563788
Purified anti-human CD314 (NKG2D) Antibody	Biolegend	Cat# 320802 RRID: AB_492956
Human GITR/TNFRSF18 Antibody	R&D systems	Cat# MAB689 RRID: AB_2203992
Human LAG3 Antibody	R&D systems	Cat# AF2319 RRID: AB_416576
Human GATA3 Antibody	R&D systems	Cat# MAB26052
Human CD25 Antibody	R&D systems	Cat# AF-223-NA RRID: AB_354408
Purified anti-human CXCR3 (Maxpar® Ready) Antibody	Fluidigm	Cat# 3163004B
Purified anti-human CCR6 (Maxpar® Ready) Antibody	Fluidigm	Cat# 3141003A
Anti-Human CD45 (HI30)-89Y	Fluidigm	Cat# 3089003B
TIGIT Monoclonal Antibody (MBSA43), Functional Grade, eBioscience™	Thermo	Cat# 16-9500-82 RRID: AB_10718831
FOXP3 Monoclonal Antibody (PCH101), Functional Grade, eBioscience™	Thermo	Cat# 14-4776-82 RRID: AB_467553
APC/Cyanine7 anti-human CD279 (PD-1) Antibody	Biolegend	Cat# 367416 RRID: AB_2616744
APC anti-human CD279 (PD-1) Antibody	Biolegend	Cat# 367406 RRID: AB_2566067
PE/Cyanine7 anti-human CD223 (LAG-3) Antibody	Biolegend	Cat# 369208 RRID: AB_2629835
PerCP/Cyanine5.5 anti-human CD223 (LAG-3) Antibody	Biolegend	Cat# 369216 RRID: AB_2910413
APC anti-human CD3 Antibody	Biolegend	Cat# 300439 RRID: AB_2562045
PE/Cyanine7 anti-human CD3 Antibody	Biolegend	Cat# 300420 RRID: AB_439781
APC/Cyanine7 anti-human CD4 Antibody	Biolegend	Cat# 317418 RRID: AB_571947
PE anti-human CD8 Antibody	Biolegend	Cat# 303804 RRID: AB_2860786
APC anti-human CD25 Antibody	Biolegend	Cat# 356110 RRID: AB_2561977
PE/Cyanine7 anti-human CD69 Antibody	Biolegend	Cat# 310912 RRID: AB_314847
APC anti-human CD69 Antibody	Biolegend	Cat# 310910 RRID: AB_314845
PE anti-human CD69 Antibody	Biolegend	Cat# 310906 RRID: AB_314841
PE anti-human NKG2D Antibody	Biolegend	Cat# 320806 RRID: AB_492960
APC anti-human MICA/B Antibody	Biolegend	Cat# 320908 RRID: AB_493196
Human ULBP-2/5/6 APC-conjugated Antibody	R&D systems	Cat# FAB1298A RRID: AB_2257142
Human Mesothelin APC-conjugated Antibody	R&D systems	Cat# FAB32652A RRID: AB_2298058
APC Mouse IgG1, κ Isotype Ctrl Antibody	Biolegend	Cat# 400120 RRID: AB_2888687
APC anti-mouse IgG2a Antibody	Biolegend	Cat# 407110 RRID: AB_2561754
SMAD2/3(D7G7) XP® Rabbit mAb	Cell Signaling Technology	Cat# 8685 RRID: 10891619
Phospho-SMAD2(Ser465/467)/SMAD3 (Ser423/425) (D27F4) Rabbit mAb	Cell Signaling Technology	Cat# 8828 RRID: 2631089
DRP1 (D6C7) Rabbit mAb	Cell Signaling Technology	Cat# 8570 RRID: AB_10950498
Phospho-DRP1 (Ser616) (D9A1) Rabbit mAb	Cell Signaling Technology	Cat# 4494 RRID: AB_11178659
OPA1 (D7C1A) Rabbit mAb	Cell Signaling Technology	Cat# 67589 RRID: AB_2799728
MFF (E5W4M) XP® Rabbit mAb	Cell Signaling Technology	Cat# 84580 RRID: AB_2799819
SMAD4 (D3R4N) XP® Rabbit mAb	Cell Signaling Technology	Cat# 46535 RRID: AB_2736998
SMAD4 Polyclonal antibody	Proteintech	Cat# 10231-1-AP RRID: AB_2193323
α-Smooth Muscle Actin (D4K9N) XP® Rabbit mAb	Cell Signaling Technology	Cat# 19245 RRID: AB_2734735
Cleaved Caspase-3 (Asp175) (5A1E) Rabbit mAb	Cell Signaling Technology	Cat# 9664 RRID: 10828837
CD8α (C8/144B) Mouse mAb	Cell Signaling Technology	Cat# 70306 RRID: AB_2799781
Anti-CD4 antibody	Abcam	Cat# ab133616 RRID: AB_2750883
Goat Anti-Rabbit IgG H&L (Alexa Fluor® 568)	Abcam	Cat# ab175471 RRID: AB_2576207
Goat Anti-Mouse IgG H&L (Alexa Fluor® 647)	Abcam	Cat# ab150115 RRID: AB_2687948

**Biological samples**		

Patient-derived xenografts (PDX)	Jiang. et al.,^26^ Qin. et al.^46^	N/A
Heathy PBMCs	Guangzhou Tianhe Noah Biological Engineering Co., LTD	NA

**Chemicals, peptides, and recombinant proteins**		

TGF beta 1 Protein, Human, Rhesus, Cynomolgus, Canine, Recombinant	SinoBiological	CAS number: 10804-HNAC
TMRM	MedChemExpress	Cat# HY-D0984
MitoTracker Green	Yeasen	Cat# 40742ES50
MitoTracker Deep Red	Yeasen	Cat# 40743ES50
GenCRISPR™ Cas9 v1.2	GenScript	Cat# Z03702-1
Seahorse XF Cell Mito Stress Test Kit	Agilent	Cat# 103010-100

**Critical commercial assays**		

Human Granzyme B Precoated ELISA Kit	DAKEWE	Cat# 1118503
Human IFN-γ Precoated ELISA Kit	DAKEWE	Cat# 1110003
Human IL-2 Precoated ELISA Kit	DAKEWE	Cat# 1110203
Human TGFβ1 Precoated ELISA Kit	DAKEWE	Cat# 1117102
Human PRF1 Precoated ELISA Kit	Bioswamp	Cat# HM10300
MACS Pan T cell Isolation Kit	Miltenyi Biotec	Cat# 130-096-535
MACS GMP T cell TransAct	Miltenyi Biotec	Cat# 170-076-156

**Deposited data**		

Original data of the bulk RNAseq	This paper	GSA-human: HRA001397
Original western blot images	This paper	https://doi.org/10.17632/n7htzcxmw4.1 Links: https://data.mendeley.com/drafts/n7htzcxmw4/1

**Experimental models: Cell lines**		

Huh7	Procell	Cat# CL-0120
HepG2	Procell	Cat# CL-0103
SK-Hep-1	Procell	Cat# CL-0212
AsPc-1	Procell	Cat# CL-0027
Hela	Procell	Cat# CL-0292
Jukrat	Procell	Cat# CL-0315

**Experimental models: Organisms/strains**		

Mouse: NSI, NOD-SCID; IL2rg−/−	Guangzhou Institutes of Biomedicine and Health, Chinese Academy of Sciences, Guangzhou, China	N/A

**Oligonucleotides**		

GZMB target gRNA: 5′-TGTCTGCCCTGGCTTCACCT-3′	GenScript	Cat# C080C988G0
IFNG target gRNA: 5′-AAAGAGTGTGGAGACCATCA -3′	GenScript	Cat# C5095GJ130
ctrl target gRNA: 5′-CCGGGTCTTCGAGAAGACCT-3-3′	GenScript	Cat# C2639HE050

**Recombinant DNA**		

Anti-TGFβ scFv-CD28^−^CD3z-2A-eGFP	This paper	N/A
Anti-TGFβ scFv-CD28^−^CD3z-TLR2-2A-eGFP	This paper	N/A
Anti-CD19 scFv-CD28^−^CD3z-TLR2-2A-eGFP	This paper	N/A
Anti-GPC3 scFv-CD28^−^CD3z-TLR2-2A-eGFP	This paper	N/A
Anti-MSLN scFv-CD28^−^CD3z-TLR2-2A-eGFP	This paper	N/A
DnTGFβRII-2A-eGFP	This paper	N/A
Anti-TGFβ scFv-mCD28-mCD3z-mTLR2-2A-eGFP	This paper	N/A
Anti-CD19 scFv-mCD28-mCD3z-mTLR2-2A-eGFP	This paper	N/A

**Software and algorithms**		

FlowJo v10	TreeStar	https://www.flowjo.com/solutions/flowjo/downloads
Prism 9.0	GraphPad	https://www.graphpad.com/scientificsoftware/prism/
ImageJ	National Institutes of Health	https://imagej.en.softonic.com
BioRender	BioRender	https://www.biorender.com/
BGI Dr. Tom	BGI	https://biosys.bgi.com/
iDEP	iDEP	http://bioinformatics.sdstate.edu/idep/

### Experimental model and study participant details

#### Primary human T lymphocytes

Healthy PBMC donors provided informed consent for using their samples for research purposes, and all procedures were approved by the Research Ethics Board of the Guangzhou Institutes of Biomedicine and Health, Chinese Academy of Sciences (GIBH, CAS). T cells from eight donors were used in this study; donor 1 is a 54-year-old male; donor 2 is a 45-year-old female; donor 3 is a 52-year-old female; donor 4 is a 29-year-old male; donor 5 is a 37-year-old female; donor 6 is a 50-year-old male; donor 7 is a 50-year-old female; donor 8 is a 50-year-old female.

#### Cell lines

Cell lines, including Huh7, HepG2 and SK-Hep-1 (human HCC cell lines), AsPc-1 (a human pancreatic cancer cell line), HeLa (a human cervical cancer cell line), and HEK-293T (a human kidney cell line), were maintained in Dulbecco’s modified Eagle’s medium (DMEM) (Gibco, Grand Island, NY, USA). Jurkat cells (a human acute T cell leukemia cell line) were maintained in RPMI-1640 medium. Media were supplemented with 10% heat-inactivated FBS (Gibco, Grand Island, NY, USA), 10 mM HEPES, 2 mM glutamine (Gibco, Grand Island, NY, USA) and 1% penicillin/streptomycin (Gibco, Grand Island, NY, USA). All cells were cultured at 37°C in an atmosphere of 5% carbon dioxide. Cell line identities were confirmed by STR sequencing.

#### Xenograft models and *in vivo* assessment

Animal experiments were performed in the Laboratory Animal Center of GIBH, and all animal procedures were approved by the Animal Welfare Committee of GIBH. All protocols were approved by the relevant Institutional Animal Care and Use Committee (IACUC). NSI mice^27^ were maintained in specific pathogen-free (SPF)-grade cages and were provided autoclaved food and water. Direct injection of 2 × 10^6^ indicated tumor cells in 100 μL of PBS was performed to establish subcutaneous (flank) tumors. When the tumor volume was approximately 1.0 cm^3^, tumors were diced into 20–50 mm^3^ pieces. These tissues were inoculated subcutaneously into the right flank of 8-week-old male NSI mice. When the tumor volume was approximately 50–100 mm^3^, the xenografted mice were divided into different groups randomly and then labeled with indicated number. Then, 5 × 10^6^ of the indicated CAR T cells in 100 μL of PBS were adoptively transferred into the tumor-bearing mice systemically by subcutaneous injection. Murine peripheral blood was obtained by retro-orbital bleeding. The xenografted mice were randomized into different groups. The group sample size for all mouse experiments was *n* ≥ 4. Tumors were measured every 3 days with a caliper. Tumor volume was calculated using the following equation: (length × width^2^)/2. We collected murine peripheral blood from the eye vein of tumor models after ensuring the mice were completely anesthetized. Subsequently, we processed the murine peripheral blood to remove erythrocytes and increase the proportion of T cells. Flow cytometry was used to monitor the proportion of CAR T cells in murine peripheral blood once per week.

### Method details

#### Isolation, transduction, and expansion of primary human T lymphocytes

For all preclinical experiments in this study, PBMCs were isolated from healthy adult donors using Lymphoprep (Catalog#07851, Stem Cell Technologies, Vancouver, Canada). T cells were negatively selected from PBMCs using EasySep Human Naive Pan T cell Isolation Kit (Catalog#17961 130-096-535, Stem Cell Technologies, Vancouver, Canada) and activated with 10 μL MACS T cell TransAct (130-111-160, Miltenyi Biotec, Bergisch Gladbach, Germany) at a bead-to- cell ratio of 1:1 and a density of 1 × 10^6^ cells/ml for one day in T551-H3 (Takara, Japan) medium supplemented with 5% heat-inactivated fetal bovine serum (FBS), 500 U/ml recombinant human IL-2, 10 mM HEPES, 2 mM glutamine and 1% penicillin/streptomycin. CD3^+^ CAR T cells were transduced with the CAR-expressing lentiviral vectors for 24 h without ablating endogenous TGFβRII.

#### The generation of CD4^+^ and CD8^+^ CAR T cells

Firstly, CD3^+^ T cells were negatively selected from PBMCs using EasySep Human Naive Pan T cell Isolation Kit (Catalog#17961 130-096-535, Stem Cell Technologies, Vancouver, Canada). Then CD4^+^ T cells or CD8^+^ T cells were positively selected from CD3^+^ T cells using Human CD4^+^ T cell isolation kit (130-096-533, Miltenyi Biotec, Bergisch Gladbach, Germany) or Human CD8^+^ T cell isolation kit (130-096-495, Miltenyi Biotec, Bergisch Gladbach, Germany). These T cells were activated with 10 μL MACS T cell TransAct (130-111-160, Miltenyi Biotec, Bergisch Gladbach, Germany) at a bead-to-cell ratio of 1:1 and a density of 1 × 10^6^ cells/ml for one day in T551-H3 (Takara, Japan) medium supplemented with 5% heat-inactivated fetal bovine serum (FBS), 500 U/ml recombinant human IL-2, 10 mM HEPES, 2 mM glutamine and 1% penicillin/streptomycin. CD4^+^ and CD8^+^ CAR T cells were transduced with the CAR-expressing lentiviral vectors for 24 h. We also mixed the CD4^+^ and CD8^+^ CAR T cells a ratio of 1:1 to obtain CD3^+^ CAR T cells before injecting them into tumor models.

#### Flow cytometry and cell sorting

Flow cytometric analysis was performed on a FACS Canton Ⅱ or FACS Fortessa (BD, USA). Fluorescence-activated cell sorting (FACS) was performed on a FACS Aria II platform (BD, USA) or MoFlo Astrios (Beckman, USA). Surface staining for flow cytometry and cell sorting was performed by pelleting cells and resuspending them in 50 μL of FACS buffer (2% FBS in PBS) with antibodies for 30 min at 4°C in the dark. Cells were washed once in FACS buffer before resuspension. PD-1, LAG-3, CD3, CD4, CD8, CD25, NKG2D and CD69 antibodies were purchased from Biolegend (CA, USA). The antibodies used in this research are listed in key resources table.

#### Protein isolation and immunoblotting

SMAD2/3 (clone D7G7) rabbit mAb, phospho-SMAD2 (S465/467)/SMAD3 (S423/425) (clone D27F4) (pSMAD2/3) rabbit mAb, SMAD4 (clone D3R4N) rabbit mAb, DRP1 (clone D6C7) rabbit mAb, phospho-DRP1(Ser616) (pDRP1^S616^) rabbit mAb (clone D9A1), phospho-DRP1^S616^ rabbit polyclonal antibody (#3455), OPA1 (D7C1A) rabbit mAb and MFF (E5W4M) XP rabbit mAb were purchased from Cell Signaling Technology (Boston, USA). SMAD4 polyclonal antibody (Cat No. 10231-1-AP) and β-actin mAb (Cat No. 66009-1-Ig) were purchased from Proteintech Group (Chicago, USA). Cells were lysed with RIPA buffer containing protease inhibitors (1 mM phenylmethylsulfonyl fluoride (PMSF), 10 mg/L aprotinin, and 10 mg/L leupeptin) (Pierce, Rockford, Illinois, USA), and proteins were quantified using a BCA Protein Assay kit (Pierce, Rockford, IL, USA). Whole-cell lysates were separated by SDS-PAGE, transferred to a PVDF (Immobilon-P; Millipore) membrane and then subjected to immunoblotting with the indicated antibodies using the ECL detection system. Images were taken using a G6000 plus Imaging System (BLT, Guangzhou, China). The antibodies used in this research are listed in key resources table.

#### Co-immunoprecipitation (Co-IP)

A total of 4 × 10^7^ CD4^+^ T cells were lysed in 800 μL IP lysis buffer (150 mM KCl, 1% Triton X-100, 50 mM Tris-HCl pH7.6, 1 mM EDTA, 10% glycerol, 1 mM PMSF, 10 mg/L aprotinin, and 10 mg/L leupeptin), and cleared cell lysates were incubated with 10 μL Protein A/G beads (88802) (Thermo Scientific, USA) and the appropriate antibody (5–10 μg) overnight at 4°C. Following incubation, the resin was washed three times with IP wash buffer (150 mM KCl, 0.1% Triton X-100, 50 mM Tris-HCl pH7.6, 1 mM EDTA, 1 mM PMSF), and protein samples were eluted by boiling in 1 × SDS sample buffer (30 μL) for western blot analysis.

#### *In vitro* killing assays

Huh7-GFP-2A-Luciferase (Huh7-GL) cells and AsPc-1-GL were incubated with CAR T cells at the indicated ratio in triplicate in U-bottomed 96-well plates. Target cell viability was monitored 24 or 72 h later by adding the substrate D20 luciferin (potassium salt, 100 μL/well) (Cayman Chemical, Michigan, USA) at 150 μg/mL. Background luminescence was negligible (<1% of the signal from wells containing only target cells). The cytotoxicity percentage (killing %) was calculated as (blank signal-experimental signal)/blank signal × 100%.^59^

#### Cytokine release assays

Supernatant from each sample was collected after endpoint killing assays or longitudinal killing analysis and stored at −20°C until further analysis. Cytokine concentrations were quantified by ELISA kits according to the manufacturer’s instructions. All ELISA kits used in this study are listed in key resources table.

#### Metabolic assays

For measurement of mitochondrial membrane potential, fresh isolated T cells were stained with 100 nM TMRM (HY-D0984, MedChemExpress, USA) in culture medium for 10 min at 37°C. After washing three times with PBS, cells were loaded with surface markers and processed for flow cytometry. For analysis of the OCR (in pmol/min), fresh isolated T cells (200,000 cells well^−1^) were plated on Cell-Tak (BD Biosciences) pretreated Seahorse plates in XF media (25 mM glucose, 2 mM glutamine and 1 mM pyruvate) and analyzed using the Seahorse XF^e^24 metabolic extracellular flux analyzer (Agilent Technologies). Basal OCR was measured for 30 min. Cells were cocultured with 2 mM oligomycin, 1.5 mM FCCP, and 1 mM each of rotenone and antimycin A (all drugs were from Agilent Technologies), to measure maximum respiration and excess respiratory capacity. The CD4^+^ or CD8^+^ T cells were sorted by MACS human CD4 microbeads (130-045-101) or human CD8 microbeads (130-045-201), while CD4^+^ CAR^+^ T28zT2 or G28zT2 T cells were sort by FACS Aria. The purity of CD4^+^, CD8^+^, and CD4^+^ CAR^+^ T cells were >90% for seahorse analysis.

#### Immunohistochemistry (IHC) assays

Anti-CD8α mouse mAb (clone C8/1448) and a-Smooth Muscle Acitn (α-SMA) (clone D4K9N) rabbit mAb were purchased from Cell Signaling Technology (Boston, USA). Anti-CD4 rabbit mAb (clone EPR6855) was purchased from Abcam (Cambridge, UK). IHC assay was performed based on a microwave-enhanced avidin-biotin staining method as previously described. Formalin-fixed, paraffin-embedded tumor tissue slides from 1928zT2, T28zT2, or G28zT2 group were deparaffinized using xylene and graded ethyl alcohol and then rinsed in water. Antigen retrieval was performed by boiling the slides in 0.01 M citrate buffer in a microwave oven for 5 min and cooling at room temperature. The slides were then incubated with 0.1% Tween 20 in PBS for 5 min. After the quenching of endogenous peroxides with 3% H_2_O_2_ in methanol, the slides were subjected to sequential treatments in a humidified chamber. The slides were blocked with 3% BSA for 30 min at room temperature, and then incubated with CD8α mouse mAb (dilution 1:100) and CD4 rabbit mAb (dilution 1:100) overnight at 4°C. The slides were subsequently incubated with the secondary rabbit-antibody for 30 min at room temperature. Next, the slides were stained successively with DAB dye for 5 min at room temperature, counterstained with hematoxylin, and coverslipped. The percentages of CD4^−^and CD8-positive cells were quantified as the average from four fields in each slide. Images were obtained under a microscope (Leica DMI6000B, Leica Microsystems, Wetzlar, Germany). The antibodies used in this research are listed in key resources table.

#### Immunofluorescence staining

Anti-Cleaved Caspase-3 (CC3) rabbit mAb (clone 5A1E) was purchased from Cell Signaling Technology (Boston, USA). Anti-CD4 rabbit mAb (clone EPR6855) was purchased from Abcam (Cambridge, UK). Purified anti-human CD314 (NKG2D) Antibody (clone 1D11) was purchased from Biolegend (CA, USA). Formalin-fixed, paraffin-embedded tumor tissue slides from 1928zT2, T28zT2 or G28zT2 group were deparaffinized using xylene and graded ethyl alcohol and then rinsed in water. Antigen retrieval was performed by boiling the slides in 0.01 M citrate buffer in a microwave oven for 5 min and cooling at room temperature. The slides were then incubated with 0.5% Triton X-100 in PBS for 30 min. The slides were blocked with 3% BSA for 30 min at room temperature, and then were incubated with CC3 mAb (dilution 1:100) overnight at 4°C, and cleared in PBST buffer (0.1% Tween 20). The slides were incubated with Goat Anti-Rabbit IgG H&L (Alexa Fluor 568) and/or Goat Anti-Mouse IgG H&L (Alexa Fluor 647) applied 1:1000 in 3% BSA for 90 min at room temperature, followed by mounting with DAPI-containing mounting medium. Fluorescence images were acquired with a Zeiss LSM 710 NLO scanning confocal imaging workstation (Oberkochen, Germany). The antibodies used in this research are listed in key resources table.

#### Histological analysis

Organ or tissue samples from mice were first fixed in 10% formalin to preserve their structure. Following fixation, the samples were embedded in paraffin. The embedded tissue was then sectioned at a thickness of 4 μm. Finally, the sections were stained with hematoxylin and eosin. Images of the stained sections were obtained using a microscope (Leica DMI6000B, Leica Microsystems, Wetzlar, Germany).

#### Mass cytometry sample preparation and acquisition

Cells from culture suspensions were fixed with 5 mM cisplatin in PBS (Fluidigm, USA) for 5 min on ice and then washed with PBS with 0.5% BSA and 0.02% NaN_3_. The cells were suspended in Fc receptor blocking mixture, incubated for 20 min on ice, and subsequently stained with a metal-labeled mAb cocktail against cell-surface molecules for 30 min on ice. The antibodies were either purchased preconjugated from Fluidigm or conjugated in-house using mass cytometry antibody conjugation kits (Fluidigm, CA, USA) according to the manufacturer’s instructions. The antibodies included CD45RA, CD45RO, CD3, CD8, CD4, CD197, CD56, CX3CR1, NKG2D, CD27, CD28, CD69, TIM-3, PD-1, CTLA-4, IL7R, TCF1, T-bet, GITR, LAG3, GATA3, CD25, CXCR3, CCR6, TIGIT, FOXP3 and CD45. The cells were then washed and stained with 1 mL of 1: 1000 191/193Ir DNA intercalator (Fluidigm) diluted in Fix and Perm (Fluidigm) at 4°C overnight. After treatment with fixation/permeabilization buffer (Thermo Fisher, USA), the cells were further incubated with a metal-labeled mAb cocktail against intracellular proteins. Immediately before acquisition, the cells were washed once with PBS with 0.5% BSA and 0.02% NaN_3_, once with ddH_2_O, and then suspended in ddH_2_O containing bead standards (Fluidigm, USA) at approximately 1 × 10^6^ cells per mL. Samples were acquired on a CyTOF (Fluidigm, CA, USA) at an event rate of <300 events/second.^60^ The antibodies used in this research are listed in key resources table.

#### Bulk RNA-seq

mRNA extracted from purified T cells was prepared according to the TruSeqTM RNA Sample Preparation Guide, and sequencing was performed on a BGISEQ-500^61^^,^^62^ (BGI, Wuhan, China). Sequenced reads were trimmed for adaptor sequences and masked for low-complexity or low-quality sequences. The number of raw reads mapped to genes was calculated by RSEM (rsem-1.2.4), and the sample results were combined and normalized by EDAseq (1.99.1). Gene expression fold changes were calculated using normalized raw reads. The downstream analysis used glbase scripts.

#### Generation of Granzyme B or IFN-γ knockout T28zT2 (T28zT2 sg*GZMB* or T28zT2 sg*IFNG*) CD4 T cells

Cas9/sgRNA ribonucleoprotein (RNP) was prepared immediately before experiments by incubating 20 μM Cas9 protein with 20 μM sgRNA (sgctrl: 5′-CCGGGTCTTCGAGAAGACCT-3′,^63^ sg*GZMB*: 5′-TGTCTGCCCTGGCTTCACCT-3’; sg*IFNG*: 5′-AAAGAGTGTGGAGACCATCA-3′, designed by CCTop–CRISPR/Cas9 target online predictor at https://www.cos.uni-heidelberg.de/en) at a 1:1 ratio in Human T cell Nucleofector buffer at 37°C for 15 min to a final concentration of 10 μM per 1 × 10^7^ T cells.^64^ For lentivirus transduction of T28zT2 CAR, on post-activation day 1, CD4^+^ T cells were transfected with lentivirus at an MOI of 10. Twelve hours after transduction, T cells were electroporated with Cas9-sgRNA mixture. Fresh medium was added every 2 days to maintain cell density within the range of 1 × 10^6^ cells/ml.

### Quantification and statistical analysis

Statistical significance was determined using Student’s t test (two groups) or ANOVA with Tukey’s multiple comparison test (three or more groups). Kaplan-Meier survival curves of *in vivo* experiments were analyzed using log rank. All statistical analyses were performed using Prism version 9.0 (GraphPad, Inc., San Diego, CA, USA). P > 0.05 = NS, ∗*p* < 0.05, ∗∗P ≤ 0.01, ∗∗∗P ≤ 0.001 and ∗∗∗∗P ≤ 0.0001 were considered statistically significant.

### Additional resources

A clinical trial related to this study was registered on https://clinicaltrials.gov/study/NCT03198052. Its registration number is NCT03198052.

## Supplemental information

Document S1. Figures S1–S6

Table S1. Upregulated DEGs of CAR+CD4^+^ T cells, related to Figure 2

Table S2. Downregulated DEGs of CAR+CD4^+^ T cells, related to Figure 2

Table S3. Upregulated DEGs of tumor-infiltrating CD3^+^ cells, related to Figure 2

Table S4. Downregulated DEGs of tumor-infiltrating CD3^+^ cells, related to Figure 2

Document S2. Article plus supplemental information
