# Supplementary material for: CD4+ anti-TGF-β CAR T cells and CD8+ conventional CAR T cells exhibit synergistic antitumor effects
Source: Cell Rep Med. 2025 Mar 18;6(3):102020. doi: 10.1016/j.xcrm.2025.102020 (PMC11970399; doi:10.1016/j.xcrm.2025.102020)
Supplement: Document S1. Figures S1–S6 [file mmc1.pdf]

Cell Reports Medicine, Volume *6*

Supplemental information

CD4$^+$ anti-TGF-β CAR T cells and CD8$^+$ conventional CAR T cells exhibit synergistic antitumor effects

Diwei Zheng, Le Qin, Jiang Lv, Meihui Che, Bingjia He, Yongfang Zheng, Shouheng Lin, Yuekun Qi, Ming Li, Zhaoyang Tang, Bin-Chao Wang, Yi-Long Wu, Robert Weinkove, Georgia Carson, Yao Yao, Nathalie Wong, James Lau, Jean Paul Thiery, Dajiang Qin, Bin Pan, Kailin Xu, Zhenfeng Zhang, and Peng Li

Figure. S1

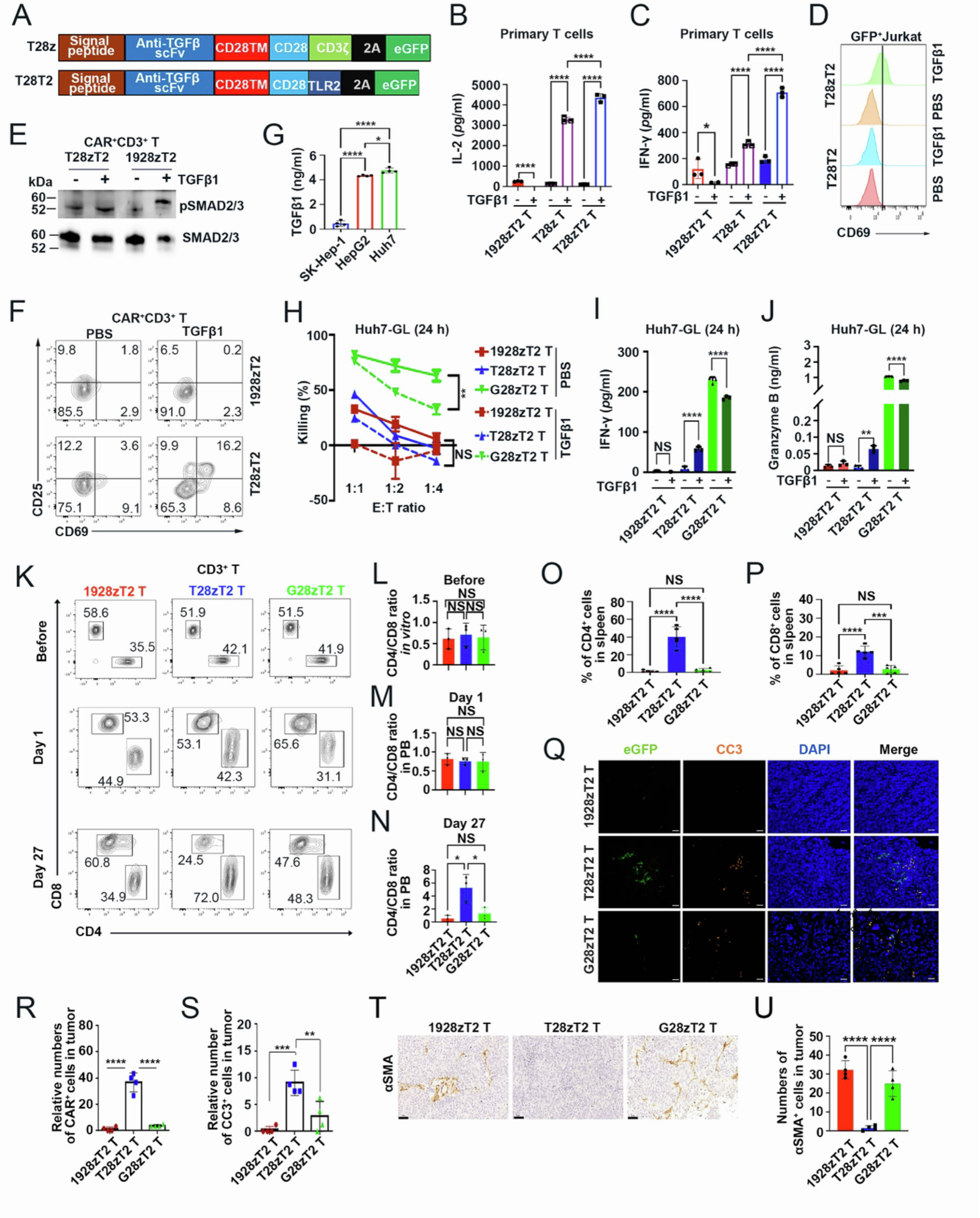

Figure S1. Anti-TGFβ CAR T cells secreted cytolytic cytokines *in vitro* and promoted T cell expansion *in vivo,* Related to Figure 1

(A) T28T2 CAR vector consisted of an anti-TGFβ scFv, a human CD8 leader signal peptide, CD28, and a TLR2 signaling domain along with eGFP using 2A. T28z CAR vector consisted of an anti-TGFβ scFv (US20140127230A1), a human CD8 leader signal peptide, CD28, and a CD3ζ signaling domain along with eGFP using 2A.

(B-C) IL-2 and IFN-γ expression of 5×10^5 T28zT2 T cells, T28z T cells or 1928zT2 T cells with or without TGFβ1 treatment (10 ng/ml) in culture for 72 hours. Measured by 3 independent ELISA assay experiments. Data are shown as the mean $\pm$ SEM values; one-way ANOVA with Tukey's multiple comparisons test; *P < 0.05, ****P $\leq$ 0.0001.
(D) Flow cytometric analysis of human CD69 expression in 1×10^5 T28zT2 Jurkat cells (GFP% > 95%) or T28T2 Jurkat cells (GFP% > 95%) with or without TGFβ1 treatment (10 ng/ml) in culture for 24 hours. Representative data shown.
(E) Immunoblot analysis of $pSMAD2^{S465/467}$/$pSMAD3^{S423/425}$ (pSMAD2/3; top) and SMAD2/3 (bottom) in CAR^+CD3^+ T28zT2 and CAR^+CD3^+ 1928zT2 T cells with or without TGFβ1 treatment (10 ng/ml) for 24 hours in culture.
(F) Flow cytometric analysis of human CD25 and CD69 expression in 4×10^5 CAR^+ (GFP^+) $CD3^+$ T28zT2 T cells or 1928zT2 T cells with or without TGFβ1 treatment (10 ng/ml) for 24 hours in culture.
(G) TGFβ1 protein levels of 3×10^5 SK-Hep-1, HepG2 and Huh7 cells were measured by ELISA assay after 48 hours of culture in 6-well plates. HCC cell supernatants were harvested and analyzed with a multiplex immunoassay to determine cytokine concentrations. Data are shown as the mean $\pm$ SD values; one-way ANOVA with Tukey's multiple comparisons test; *P < 0.05; ****P $\leq$ 0.0001.
(H) The percentage of Huh7 cells whose lysis was induced by 1928zT2, T28zT2, or G28zT2 T cells with or without TGFβ1 treatment (10 ng/ml) after 24 hours. Data are shown as the mean percentage of tumor cell-specific lysis $\pm$ SEM values; *n* = 3 independent experiments; two-way ANOVA with Tukey's multiple comparisons test; **P $\leq$ 0.01 (G28zT2 vs. G28zT2 treated with TGFβ1).
(I-J) Concentrations of IFN-γ (I) and Granzyme B (J) measured by ELISA assay of 1928zT2, T28zT2, and G28zT2 T cells incubated with Huh7 cells at a 1:1 effector (E): target (T) ratio in the presence of TGFβ1 or PBS in 96-well round bottom plates for 24 hours at 37 °C. Supernatants were harvested and analyzed with a multiplex immunoassay to determine cytokine concentrations. Data are shown as the mean $\pm$ SEM values; *n* = 3 independent experiments; one-way ANOVA with Tukey's multiple comparisons test; **P $\leq$ 0.01, ****P $\leq$ 0.0001.
(K-N) The CD4 and CD8 expression of 1928zT2, T28zT2 and G28zT2 T cells detected by flow cytometry of the pre-injected CAR T cells (K, Before), or murine peripheral blood shortly after infusion (K, Day 1) and at the end point (K, Day 27). (L) The CD4/CD8 T cell ratios of pre-injected CAR T cells from three different donors. (M-N) The CD4/CD8 T cell ratios of murine peripheral blood (PB) shortly after infusion (Day 1) and at the end point (Day 27). *n* = 3 mice/group. Data are shown as the mean ± SD values; one-way ANOVA with Tukey's multiple comparisons test; *P < 0.05.
(O-P) The percentages of $CD4^+$ (O) and $CD8^+$ (P) T cells in all nucleated cells from spleen of Huh7 tumor models in the 1928zT2, T28zT2, and G28zT2 groups on day 27 determined by flow cytometry (*n* = 4 mice per group). Data are shown as the mean ± SD values; one-way ANOVA with Tukey's multiple comparisons test; ***P ≤ 0.001, ****P ≤ 0.0001.
(Q-S) Immunofluorescence (IF) images of anti-cleaved Caspase-3 antibody (CC3, orange) primary staining, CAR T cells expressing GFP (green), and nuclei stained with DAPI (blue). (Q) Confocal images are representative. Scale bar, 20 μm. The frequencies of CAR^+ T cells, defined as GFP^+ cells in tumors (R), and $CC3^+$ cells in tumors (S) were calculated by ImageJ software (*n* = 4 mice per group). Data are shown as the mean $\pm$ SD values; one-way ANOVA with Tukey's multiple comparisons test; **P $\leq$ 0.01, ***P $\leq$ 0.001, ****P $\leq$ 0.0001.

(T-U) Representative images of αSMA$^+$ cells (T, brown) in Huh7 tumors from the T28zT2, G28zT2, and 1928zT2 groups on day 27. (U) ImageJ software calculated the numbers of aSMA$^+$ cells (*n* = 4 mice per group). Scale bar, 50 μm. Data are shown as the mean ± SD values; one-way ANOVA with Tukey's multiple comparisons test**;** ****P ≤ 0.0001.

Figure S2

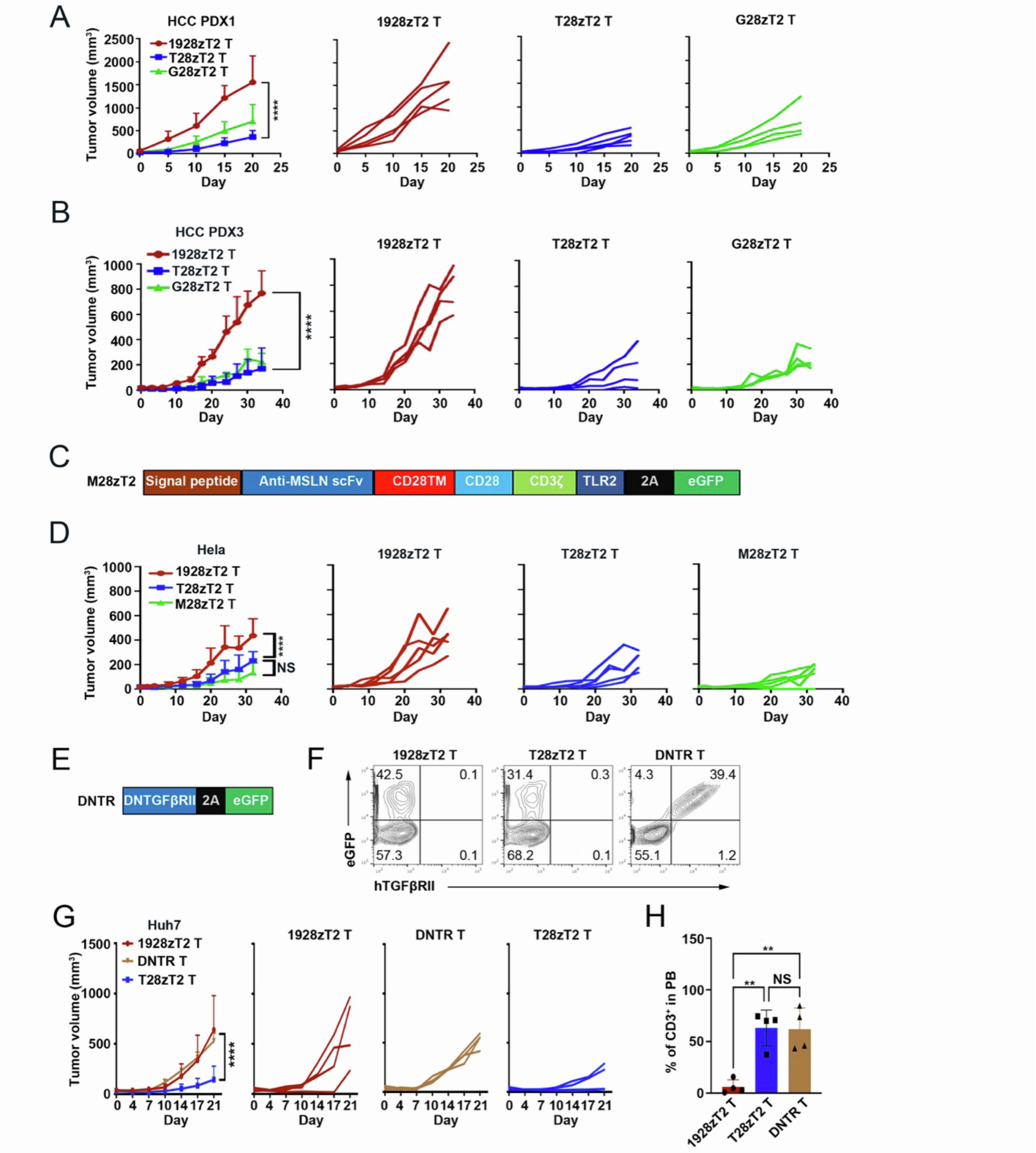

Figure S2. Anti-TGFβ CAR T cells suppressed the growth of HCC and cervical cancer *in vivo*, Related to Figure 1

(A-B) HCC PDX1 or HCC PDX3 tumors were diced into ~30 mm^3 pieces and tissue inoculated subcutaneously into the right flanks of 8-week-old male NSI mice. When the tumor volume was ~50-100 mm^3, 5×10^6 T28zT2, G28zT2, or 1928zT2 T cells were injected peritumorally (day 0). (A) Tumor growth curves for HCC PDX1 model mice (*n* = 5 mice/1928zT2 or T28zT2 group, *n* = 4 mice/G28zT2 group). (B) Tumor growth curves for HCC PDX3 model mice (*n* = 4 mice/group). Data are shown as the mean ± SD values; two-way ANOVA with Tukey's multiple comparisons test; ****P ≤ 0.0001.

(C) Anti-MSLN CAR vector (M28zT2) consisted of anti-MSLN (SS1) scFv, human CD8 leader signal

peptides, CD28, CD3ζ, TLR2 signaling domains, and eGFP using 2A.
(D) Eight-week-old male NSI mice were inoculated subcutaneously with 1×10^6 Hela cells into the right flanks. Once tumor volumes reached 1.0 cm^3, tumor tissue was diced into ~50 mm^3 pieces and transplanted into the right flanks of 8-week-old male NSI mice. A total of 5×10^6 CAR T cells were injected peritumorally once xenograft volume was ~50 mm^3 (day 0). Tumor growth curves for Hela model mice (n = 5 mice group). Data are shown as the mean $\pm$ SD values; two-way ANOVA with Tukey's multiple comparisons test; ****$P \leq 0.0001$.
(E) The DNTGFβRII vector (DNTR) consisted of the TGFβRII extracellular domain, and eGFP with 2A.
(F) Flow cytometric analysis of human TGFβRII and eGFP expression in 1928zT2, T28zT2 and DNTR T cells. Endogenous TGFβRII was not ablated in any T cells in this study.
(G) Curves showing variations in the volume of Huh7 tumors in NSI mice post infusion of T28zT2, DNTR or 1928zT2 T cells (n = 4 mice/group). Data are shown as the mean $\pm$ SD values; two-way ANOVA with Tukey's multiple comparisons test; ****$P \leq 0.0001$.
(H) The percentages of $CD3^+$ T cells in murine PB populations for the T28zT2, DNTR, and 1928zT2 groups on day 21 determined by flow cytometry. Data are shown as the mean $\pm$ SD values; one-way ANOVA with Tukey's multiple comparisons test; **$P \leq 0.01$.

Figure S3

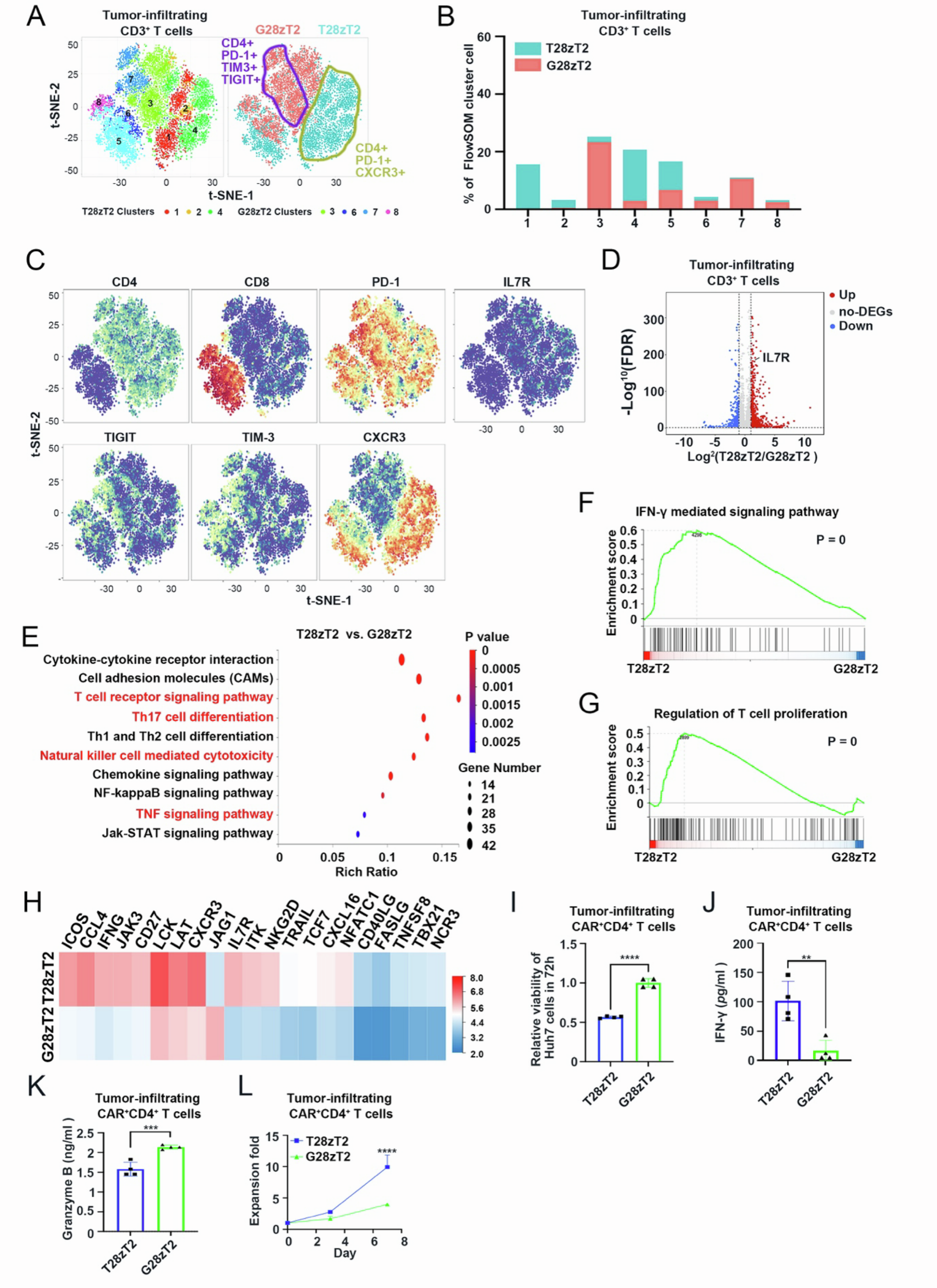

Figure S3. Tumor-infiltrating anti-TGFβ CAR T cells were resistant to exhaustion, Related to Figure 2

(A) 2D t-SNE projection of the subclusters (left) and sample distribution of tumor-infiltrating $CD3^+$ T cells from the T28zT2 group (in blue) and G28zT2 group (in red) (right).
(B) FlowSOM cluster distribution of T28zT2 group (in blue) and G28zT2 group (in green).
(C) Differences in gene expression of individual tumor-infiltrating $CD3^+$ T cells from the T28zT2 and G28zT2 groups in the t-SNE projection, selected markers include CD4, CD8, PD-1, TIGIT, TIM-3, IL7R and CXCR3.
(D) Volcano plot of DEGs showing upregulated (red) and downregulated (blue) DEGs and non-DEGs (gray) identified by RNA-seq in T28zT2 group compared to G28zT2 group. Adjustment for the false discovery rate (FDR) results in an adjusted P value called the q value. The *y*-axis shows the significance value after $-\log_{10}$ transformation of the FDR ($-\mathrm{Log}_{10}$(FDR)). The x-axis shows the fold difference threshold between the T28zT2 and G28zT2 groups (Log_2FC).
(E) Plot of the KEGG pathway enrichment analysis results comparing tumor-infiltrating $CD3^+$ T cells between the T28zT2 and G28zT2 groups. The y-axis represents pathways; the x-axis represents the amount of T28zT2 group with enrichment of the specific KEGG pathway in comparison with G28zT2 group; $P < 0.0025$.
(F-G) GSEA of the IFN-γ mediated signaling pathway (F, $P = 0$) and regulation of T cell proliferation (G, $P = 0$) in tumor-infiltrating $CD3^+$ T cells. From left to right, the genes in the rank-ordered list are enriched in the T28zT2 and G28zT2 groups.
(H) Heat map of DEGs identified in comparisons between T28zT2 group and G28zT2 group. The y-axis shows differential gene expression values expressed as log (value+1) for visualization and colored from blue to red; the x-axis shows the group (T28zT2 group or G28zT2 group). Cutoff: absolute log2 (fold change) ≥ 1; adjusted P value < 0.05.
(I-L) Tumor tissue of G28zT2 and T28zT2 groups were obtained from Huh7 tumor models at the end points (Day 27), and prepared into single-cell suspension using human tumor cell isolation kits (Miltenyi). Tumor-infiltrating CAR^+CD4^+ T cells were sorted by FACS and activated by CD3/CD28 mAbs for 48 hours before cytotoxicity, cytokine production and T cell expansion were evaluated. Tumor-infiltrating CAR^+CD4^+ T28zT2 and G28zT2 T cells were incubated with Huh7 cells at a 2:1 effector (E): target (T) ratio in 12-well round bottom plates *ex vitro* for 72 hours. (I) The relative viability of Huh7 cells with tumor-infiltrating CAR^+CD4^+ T28zT2 or G28zT2 T cells induced lysis after 72 hours *ex vitro*. Supernatants were harvested and analyzed by multiplex ELISA immunoassay to determine IFN-γ (J) and Granzyme B (K) cytokine concentrations. *n* = 4 mice/group. Data are shown as the mean ± SD values; unpaired two-tailed t test; **P ≤ 0.01, ***P ≤ 0.001, ****P ≤ 0.0001. (L) The expansion of tumor-infiltrating CAR^+CD4^+ T28zT2 and G28zT2 T cells were detected by flow cytometry at day 0, 3, and 7. Data are shown as the mean ± SD values; *n* = 4 mice/group; two-way ANOVA with Tukey's multiple comparisons test; ****P ≤ 0.0001.

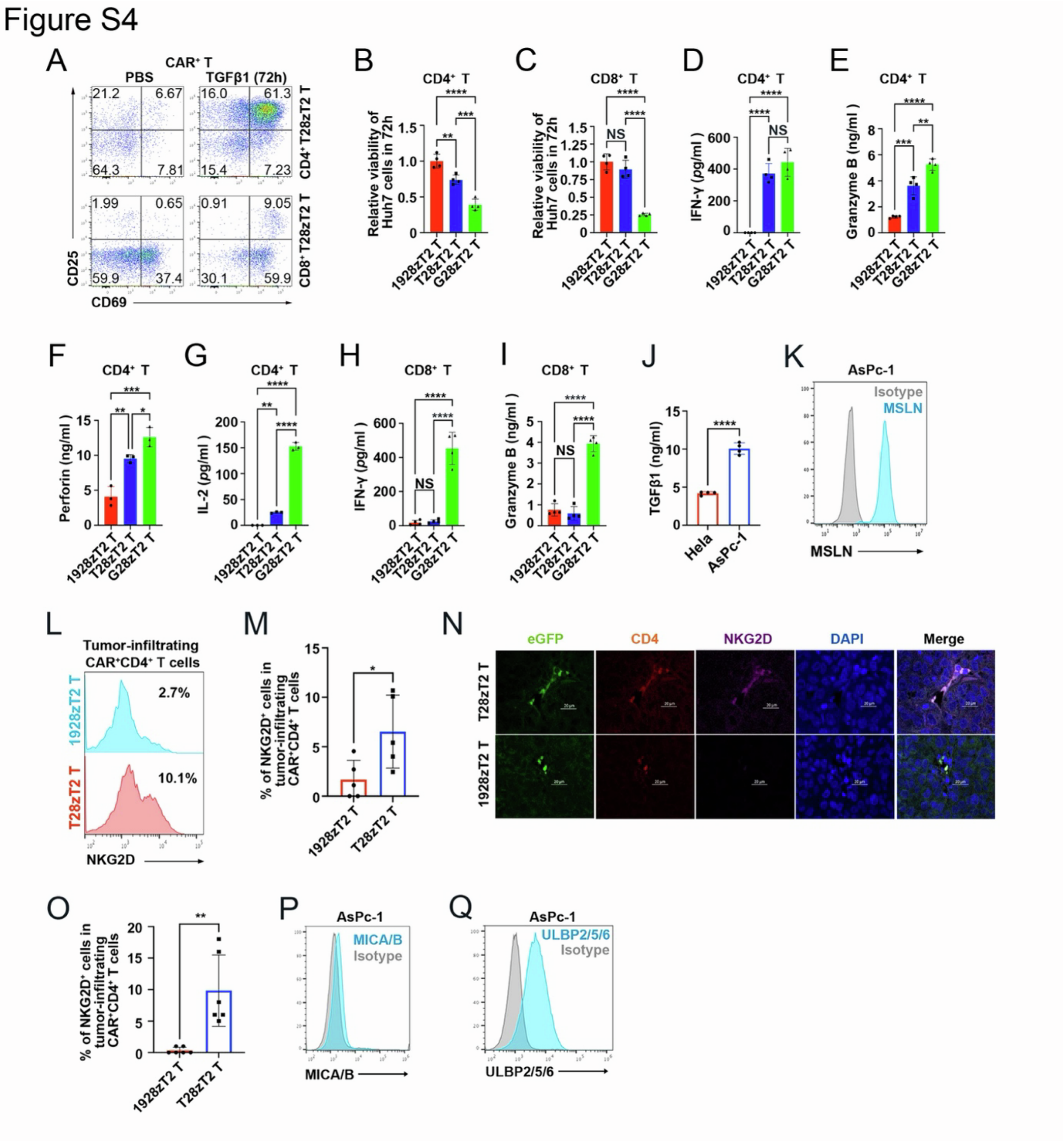

Figure S4. The cytotoxic responses of CD4⁺ anti-TGFβ CAR T cells *in vitro,* Related to Figure 3

(A) The percentages of CD25 and CD69 positivity in CD4⁺ and CD8⁺ T28zT2 T cells measured by flow cytometry after 72 hours of treatment with PBS or TGFβ1(10 ng/ml). Representative figures shown.

(B, D-G) A total of 4 × 10^5 CD4⁺ 1928zT2, T28zT2 or G28zT2 T cells were cocultured with 1 × 10^5 Huh7 cells for 72 hours. Quantification of residual tumor cells (B) and summary of IFN-γ (D), Granzyme B (E), perforin (F) and IL-2 (G) released by CD4⁺ CAR T cells. The data for Fig. S10B, S10D-E represent 4 independent experiments, and the data for Fig. S10F-G represent 3 independent experiments. Data are shown as the mean ± SEM values; one-way ANOVA with Tukey's multiple comparisons test; $*P < 0.05$, $**P \leq 0.01$, $***P \leq 0.001$, $****P \leq 0.0001$.

(C, H, I) A total of 4 × 10^5 CD8⁺ 1928zT2, T28zT2 or G28zT2 T cells were cocultured with 1 × 10^5 Huh7 cells for 72 hours. Quantification of residual tumor cells (C) and summary of IFN-γ (H) and Granzyme B (I) released by CD8⁺ CAR T cells (from 4 independent experiments); (C, H, I) Data are

shown as the mean $\pm$ SEM values; one-way ANOVA with Tukey's multiple comparisons test; ****$P \leq 0.0001$.

(J) 3×10^5 Hela or AsPc-1 cells were cultured in 6-well plates for 48 hours. Supernatants were harvested and analyzed by multiplex ELISA immunoassay to determine TGFβ1 concentrations. Data are shown as the mean ± SD values; unpaired two-tailed t test; ****$P \leq 0.0001$.

(K) The expression of MSLN in AsPc-1 cells was detected by flow cytometry. Representative figures shown.

(L-M) The percentages of $NKG2D^+$ in tumor-infiltrating CAR^+CD4^+ T cells from the 1928zT2 and T28zT2 groups determined by flow cytometry on Day 28. Data are shown as the mean $\pm$ SD values; unpaired two side t-test; *$P < 0.05$.

(N-O) Immunofluorescence (IF) images of anti-CD4 antibody (CD4, red) and anti-NKG2D antibody (NKG2D, purple) primary staining, CAR T cells expressing GFP (green), and nuclei stained with DAPI (blue). (N) Confocal images are representative. Scale bar, 20 μm. (O) The frequencies of CAR^+CD4^+ $NKG2D^+$ T cells in tumors were calculated by ImageJ software (6 random sights per group).

(P-Q) The expression of MICA/B (P) and ULBP2/5/6 (Q) in AsPc-1 cells detected by flow cytometry. Representative figures shown.

Figure S5

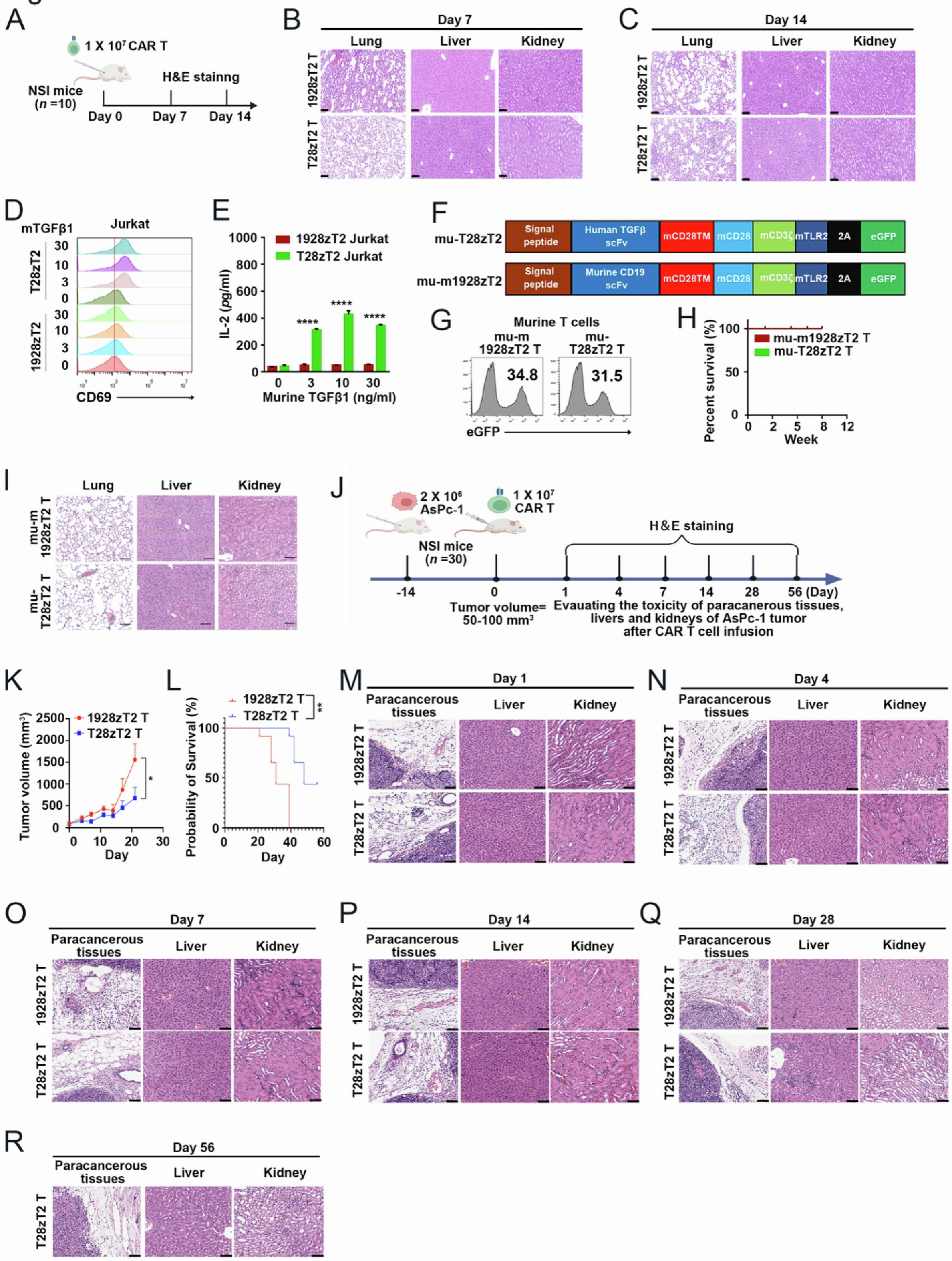
A
1 X 10^7 CAR T
NSI mice
(n =10)
H&E stainng
Day 0
Day 7
Day 14
B
Day 7
Lung
Liver
Kidney
1928zT2 T
T28zT2 T
C
Day 14
D
mTGFβ1
Jurkat
T28zT2
1928zT2
CD69
E
1928zT2 Jurkat
T28zT2 Jurkat
IL-2 (pg/ml)
Murine TGFβ1 (ng/ml)
F
mu-T28zT2
mu-m1928zT2
Signal peptide
Human TGFβ scFv
Murine CD19 scFv
mCD28TM
mCD28
mCD3ζ
mTLR2
2A
eGFP
G
Murine T cells
mu-m 1928zT2 T
mu-T28zT2 T
34.8
31.5
eGFP
H
Percent survival (%)
mu-m1928zT2 T
mu-T28zT2 T
Week
I
mu-m 1928zT2 T
mu-T28zT2 T
J
2 X 10^6 AsPc-1
1 X 10^7 CAR T
NSI mice
(n =30)
H&E staining
56 (Day)
Tumor volume= 50-100 mm³
Evauating the toxicity of paracanerous tissues, livers and kidneys of AsPc-1 tumor after CAR T cell infusion
K
Tumor volume (mm³)
1928zT2 T
T28zT2 T
Day
L
Probability of Survival (%)
1928zT2 T
T28zT2 T
Day
M
Day 1
Paracancerous tissues
N
Day 4
O
Day 7
P
Day 14
Q
Day 28
R
Day 56

Figure S5. Anti-TGFβ CAR T cells exhibited no toxicity in tumor-free mice and tumor-bearing mice, Related to Figure 3

(A) A schematic diagram of experimental design. 1 × 10^7 1928zT2 or T28zT2 T cells in 100 μl of PBS were systemically transferred into tumor-free NSI mice by subcutaneous injection (day 0). The lung, liver and kidney of tumor-free NSI mice from 1928zT2 or T28zT2 groups were harvested on day 7 and 14 after euthanization. Tissue was subjected to H&E staining and scanning. Schematic created with BioRender.com (*WF26O8AV6G*).

(B-C) Representative images of H&E staining of lung, liver and kidney tissue from tumor-free NSI mice on day 7 (B) and day 14 (C) post CAR T cell infusion. Scale bar, 100 μm.

(D-E) A total of 1 × 10^5 1928zT2 or T28zT2 Jurkat cells were treated with murine TGFβ1 (0, 3, 10 or 30 ng/ml) for 16 hours. (D) FACS analysis of CD69 expression in 1928z and T28z Jurkat cells post murine TGFβ1 treatment; (E) The human IL-2 (hIL-2) concentration in cultures of 1928zT2 and T28zT2 Jurkat cells treated with murine TGFβ1 was measured by ELISA. Data are shown as the mean $\pm$ SD values; unpaired two-tailed t test; ****$P \leq 0.0001$.

(F) Murine T28zT2 vector and m1928zT2 vector based on an anti-human TGFβ scFv (US20140127230A1) and anti-murine CD19 scFv, respectively. All vectors contained expression cassettes encoding a murine CD8 leader signal peptide; the murine CD28 transmembrane domain (TM) and endodomain, murine CD3ζ, murine TLR2 signaling domains, and eGFP with 2A.

(G) Splenic T cells from C57BL/6 mice were transduced with CAR retroviruses after incubation with CD3/CD28 mAbs for 36 hours; the percentages of transduced $eGFP^+$ cells were measured by flow cytometry.

(H) Survival curves of C57BL/6 mice after irradiation with 4.5 Gy and injection of 1 × 10^6 either mu-m1928z or mu-T28z murine CAR T cells into the tail vein ($n = 3$ mice/group).

(I) Representative images of H&E staining of lung, liver and kidney tissue from C57BL/6 mice 12 weeks post CAR T cell infusion. Scale bar, 20 μm.

(J) A schematic diagram of experimental design. NSI mice were inoculated subcutaneously with 2 × 10^6 AsPc-1 cells. Once xenograft volume was ~50 mm^3 (day 0), 1 × 10^7 1928zT2 or T28zT2 T cells in 100 μl of PBS were injected peritumorally. Tumor volumes were monitored on the indicated days (n = 30 mice/group). Paracancerous tissues, as well as liver and kidney tissues from AsPc-1 tumor-bearing mice in the 1928zT2 or T28zT2 group, were harvested on days 1, 4, 7, 14, 28 or 56, euthanizing five NSI mice per time point, or once xenograft volume exceeded 2000 mm^3. Tissue was subjected to H&E staining and scanning. Schematic created with BioRender.com (agreement number *HB27PTC4OJ*).

(K) Representative tumor volumes in AsPc-1 tumor models (n = 5 mice/group). Data are shown as the mean $\pm$ SD values; two-way ANOVA with Tukey's multiple comparisons test; *$P < 0.05$.

(L) Survival curves of AsPc-1 tumor models (n = 5 mice/group) plotted over 56-days post CAR T cell treatment. Data are shown as the mean $\pm$ SD values; statistical analysis for survival curves was performed using a log-rank test; **$P \leq 0.01$.

(M-R) Representative H&E-stained images of paracancerous tissues, liver, and kidney tissues from AsPc-1 tumor-bearing mice at 1, 4, 7, 14, 28 or 56 days post CAR T cell infusion. No mice from the 1928zT2 group survived to day 56. Scale bar, 50 μm.

Figure S6

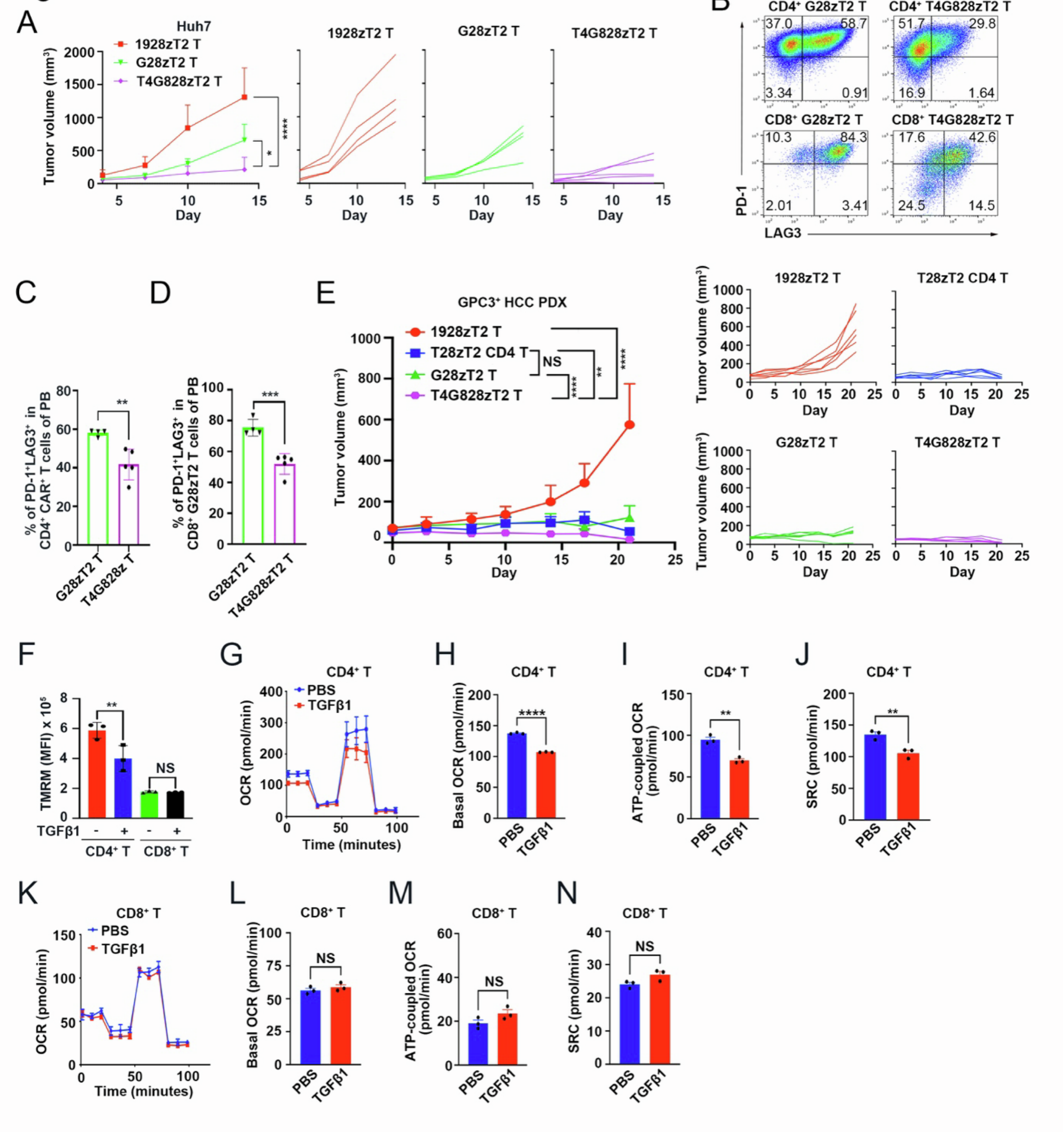

Figure S6. CD4⁺ anti-TGFβ CAR T cells partly suppressed the T cell exhaustion differentiation of CD8⁺ G28zT2 T cells *in vivo,* Related to Figures 4, 5 and 6

(A) Eight-week-old male NSI mice were inoculated subcutaneously with 2×10^6 Huh7 cells into the right flanks. Once xenograft volume was ~50 mm^3 (day 0), 5×10^6 T4G828zT2, G28zT2, or 1928zT2 T cells were injected peritumorally. (A) Tumor volumes were monitored on the indicated days (n = 4 mice/1928zT2 or G28zT2 group, n = 5 mice/T4G828zT2 group). Data are shown as the mean $\pm$ SD values; two-way ANOVA with Tukey's multiple comparisons test; *P < 0.05, ****P ≤ 0.0001.

(B-D) The percentages of PD-1⁺ LAG3⁺ among CD4⁺ CAR⁺ (B, C) and CD8⁺ CAR⁺ T cells (B, D) in PB of mice from the T4G828zT2 and G28zT2 groups on day 14 determined by flow cytometry. Data are shown as the mean $\pm$ SD values; unpaired two side t-test; **P ≤ 0.01, ***P ≤ 0.001.

(E) HCC PDX tumors were diced into ~30 mm^3 pieces and eight-week-old male NSI mice were

inoculated subcutaneously with tissue into the right flanks. Once tumor volume was ~50-100 mm^3 (day 0), 5 × 10^6 T4G828zT2, T28zT2 CD4, G28zT2, or 1928zT2 T cells were injected peritumorally. Tumor volumes were monitored on the indicated days (*n* = 6 mice/ group). Data are shown as the mean ± SD values; two-way ANOVA with Tukey's multiple comparisons test; **P ≤ 0.01, ****P ≤ 0.0001.

(F) $CD4^+$ and $CD8^+$ T cells (1 × 10^6) were activated with CD3/CD28 mAbs for 24 hours, followed by PBS or TGFβ1 (10 ng/ml) treatment for 16 hours. The level of TMRM (PE) in $CD4^+$ and $CD8^+$ T cells in the presence of TGFβ1 as detected by flow cytometry (3 independent experiments); Data are shown as the mean ± SEM values; paired two-tailed t test; **P ≤ 0.01.

(G-N) The OCR profile (G, K), basal OCR (H, L), ATP-coupled OCR (I, M), and spare respiratory capacity (SRC) (J, N) of $CD4^+$ and $CD8^+$ T cells upon treatment with TGFβ1 (10 ng/ml) (3 independent experiments). Data are shown as the mean ± SEM values; paired two-tailed t test; **P ≤ 0.01, ****P ≤ 0.0001
